# A Pill That Prints‐An Ingestible Bioprinter for Non‐Invasive Structured Bioink Deposition

**DOI:** 10.1002/advs.202512411

**Published:** 2025-09-26

**Authors:** Sanjay Manoharan, Vivek Subramanian

**Affiliations:** ^1^ Laboratory for Advanced Fabrication Technologies, Institute of Electrical and Micro Engineering École Polytechnique Fédérale de Lausanne (EPFL) Neuchâtel 2000 Switzerland

**Keywords:** gastrointestinal repair, ingestibles, in‐situ bioprinting, magnetic actuation, biofabrication, shape memory polymer

## Abstract

A foundational demonstration of a tetherless, ingestible bioprinter capable of controlled bioink deposition and patterning for potential applications in endoluminal tissue repair is presented. This ingestible microdevice, the first‐of‐its‐kind, operates without tethers or onboard electronics and is guided using external imaging and magnetic actuation. By integrating near‐infrared triggering and magnetic guidance, both applied from outside the body, the ability to guide the pill over target sites and deposit bioink in defined patterns, for ulcer coverage or hemorrhage sealing in ex vivo models is demonstrated. To enable this, the interplay is modeled and optimized between magnetic forces and stick‐slip dynamics at the tissue interface, facilitating predictable surface‐mediated navigation and patterning. In vivo studies in rabbit models showcase the technical feasibility of deployment, positioning, and controlled extrusion under fluoroscopic guidance. This work thus establishes core engineering principles for future development of non‐invasive and untethered endoluminal bioprinting systems.

## Graphical abstract

A first‐of‐a‐kind swallowable bioprinter performs in‐situ bioink deposition and patterning directly over disease sites without tethers, electronics, or invasive surgery. Combining magnetic guidance with near‐infrared triggering enables functionality within a small footprint. Novel Magnetically Modulated Stick‐Slip dynamics facilitate surface‐mediated navigation, while dispense‐and‐daub patterning spreads extruded bioink post‐deposition to create defined 2.5D patterns for intraluminal tissue repair.

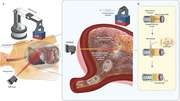

## 1 Introduction

Advances in regenerative medicine have been driven by bioprinting, tissue engineering, and biocompatible materials. Yet challenges remain where surgical intervention is too invasive or infeasible. Gastrointestinal diseases caused 2276.27 million cases and 2.56 million deaths globally in 2019,^[^ ^1^ ^]^ many linked to inflammatory bowel disease and ulcerative colitis.^[^ ^2^ ^]^ Most conventional therapies however manage the associated symptoms rather than repairing tissue, thereby highlighting opportunities for tissue engineering‐based solutions.^[^ ^3^, ^4^ ^]^ Existing tissue engineering approaches include injectable hydrogel dressings,^[^ ^5^ ^]^ decellularized extra cellular matrix (ECM) xenografts^[^ ^6^ ^]^ and invasive implantation of *ex‐situ* bioprinted extracellular matrix scaffolds,^[^ ^2^, ^7^, ^8^ ^]^ all of which require invasive surgery. To overcome these limitations, we present an ingestible bioprinter that enables in‐situ gastric bioprinting without surgery.

In‐situ bioprinting (in‐vivo or intra‐vital) directly prints biomaterials onto lesions, offering fewer infections, customizable grafts, shorter processing times, and fabrication within the healing micro‐environment, which can improve patient outcomes.^[^ ^9^, ^10^, ^11^, ^12^, ^13^ ^]^ Prior demonstrations using robotic‐arm printheads required invasive access, complex calibration and large workspaces.^[^ ^14^, ^15^, ^16^ ^]^ Addressing these limitations, researchers also explored stereolithography‐based strategies that promote in‐situ photo‐crosslinking, though their application remains confined to subcutaneous printing.^[^ ^9^, ^17^ ^]^ Recently, in‐situ bioprinters have taken diverse forms, ranging from handheld dispensers^[^ ^18^ ^]^ to continuum platforms powered by mechatronics,^[^ ^19^ ^]^ pneumatics^[^ ^20^ ^]^ and ferromagnetics^[^ ^10^ ^]^ with laparoscopic or endoscopic access. Automated platforms with closed‐loop feedback have further advanced capabilities,^[^ ^21^, ^22^ ^]^ yet challenges remain in the form of tethered configurations, anesthesia dependence and large device footprints that restrict access to deep sites (Table S1, Supporting Information). Overcoming these limitations will require miniaturization, reduced invasiveness and lower procedural complexity to enhance practitioner convenience and patient comfort.

Untethered actuation strategies are increasingly explored as solutions to these challenges, with magnetic fields emerging as a particularly promising modality. They have successfully been used in ingestible devices for biopsy^[^ ^23^ ^]^ and drug delivery^[^ ^24^, ^25^ ^]^ via pull forces from permanent magnets^[^ ^26^ ^]^ or electromagnetic coils^[^ ^27^ ^]^. These systems typically control device movement through attractive forces, torques, or rolling locomotion in fluid‐distended environments through external magnetic configurations such as robot‐mounted single permanent magnets,^[^ ^28^ ^]^ electromagnetic systems with multiple coils generating uniform or gradient fields or even a combination of coils and magnets.^[^ ^29^, ^31^ ^]^ More specifically, device‐embedded single or multiple magnets and their interaction with external fields have been used to circumvent the randomness of passive transit and advance the frameworks for multi‐modal magnetic navigation for ingestibles.^[^ ^28^, ^29^, ^30^ ^]^ Yet research has mostly focused on bulk‐fluid propulsion, considering surface‐mediated locomotion an unpredictable hindrance. For bioprinting, however, tissue contact is essential, as bioink cannot be patterned in free fluid. This reframes surface interaction from a hindrance to be avoided to a modality to be optimized, highlighting a gap in the magnetically‐driven ingestibles, one we explicitly investigate in this study.

Parallel to these bioprinting efforts, “smart capsules” have emerged for untethered gastrointestinal drug delivery including self‐orienting injectors,^[^ ^32^ ^]^ cephalopod‐inspired jetters,^[^ ^33^ ^]^ microneedle capsules^[^ ^34^ ^]^ and sampling^[^ ^31^ ^]^or refillable systems.^[^ ^35^ ^]^ While effective for systemic uptake, most rely on passive triggers (pH, enzymatic degradation, etc.) that limit spatiotemporal control (Table S2, Supporting Information), and their functions remain confined to bolus drug release without surface‐mediated navigation or structured deposition required for bioprinting.

**Figure 1:**
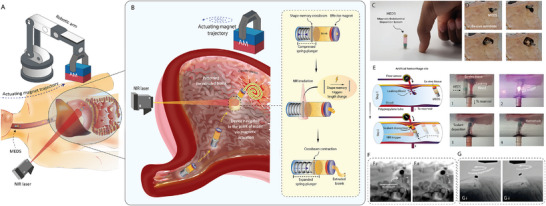
Overview of MEDS (Magnetic Endoluminal Deposition System) and its capabilities. A) Illustration of the device journey to the gastric environment, guided by magnetic trajectory control. At the defect site, NIR triggering induces bioink extrusion, and the device patterns the bioink over a gastric lesion. B) Exploded view of the device, showing its spring‐loaded plunger, bioink reservoir, and EM. C) Image of the MEDS loaded with bioink. D) Bottom view sequence of bioink dispensing and patterning on ex vivo gastric tissue. E) Illustration and in vitro demonstration of hemorrhage sealing. F) X‐ray fluoroscopic images of in vivo bioink extrusion in a rabbit model. G) Fluoroscopic visualization of in vivo patterning over the gastric wall.

At its core, a bioprinter is essentially a controlled dispenser that moves along at least two axes to pattern biomaterials. Hence, by merging the actuation principles of in‐situ bioprinters with the drug release concepts of smart capsules, one can envision a new class of device, an ingestible bioprinter. Building on that perspective, this study introduces MEDS (Magnetic Endoluminal Deposition System), the first tetherless, pill‐sized embodiment of this concept. An overview of MEDS, its components, and deployment is shown in **Figure** **1**. It integrates near infrared (NIR) triggered shape memory actuation with magnetic positioning to deposit bioink dressings directly onto soft tissue lesions, thereby improving healing outcomes (Figure 1A). A video abstract illustrating the design and functionality of the device is provided (Movie S1, Supporting Information). MEDS incorporates a bioink‐loaded milli‐scale barrel that houses a dispenser assembly, consisting of a compressed spring‐loaded plunger that releases when triggered by an externally applied NIR source (Figure 1B,C). While NIR triggers (700–900 nm) can penetrate tissue and have been applied in vivo for activating SMP‐based stents, contraceptives, and bone scaffolds,^[^ ^36^, ^37^, ^38^, ^39^, ^40^ ^]^ these devices rely on surgical implantation. This undermines the advantage of wireless, non‐invasive NIR triggering, and realizing their minimally invasive potential requires integration with tetherless in vivo positioning.

By modeling and controlling the lateral motion of the bioprinter under an external magnetic field, we achieve precise bioink patterning. After extrusion, the effector magnet (EM) is guided along X–Y trajectories using an external actuator magnet (AM) on a robotic arm (Figure 1D), enabling patterned deposition across planar and non‐planar substrates, in‐vitro hemorrhage sealing (Figure 1E), and gastric lesion dressing. In‐vivo rabbit studies confirmed successful extrusion and gastric wall patterning under fluoroscopy (Figure 1F,G), demonstrating potential for minimally invasive repair. Following printing, the device can be magnetically guided to exit orally.

## 2 Results

We demonstrate an ingestible platform enabling non‐invasive structured bioink deposition through controlled sliding/stepping locomotion at the lumen ceiling, analogous to 2.5D bioprinting techniques adapted for constrained intracorporeal environments. Our approach follows a logical progression from device design and actuation dynamics modeling, through systematic optimization and ex‐vivo validation, to in‐vivo feasibility demonstration.

### 2.1 MEDS Design and Extrusion Control

**Figure 2:**
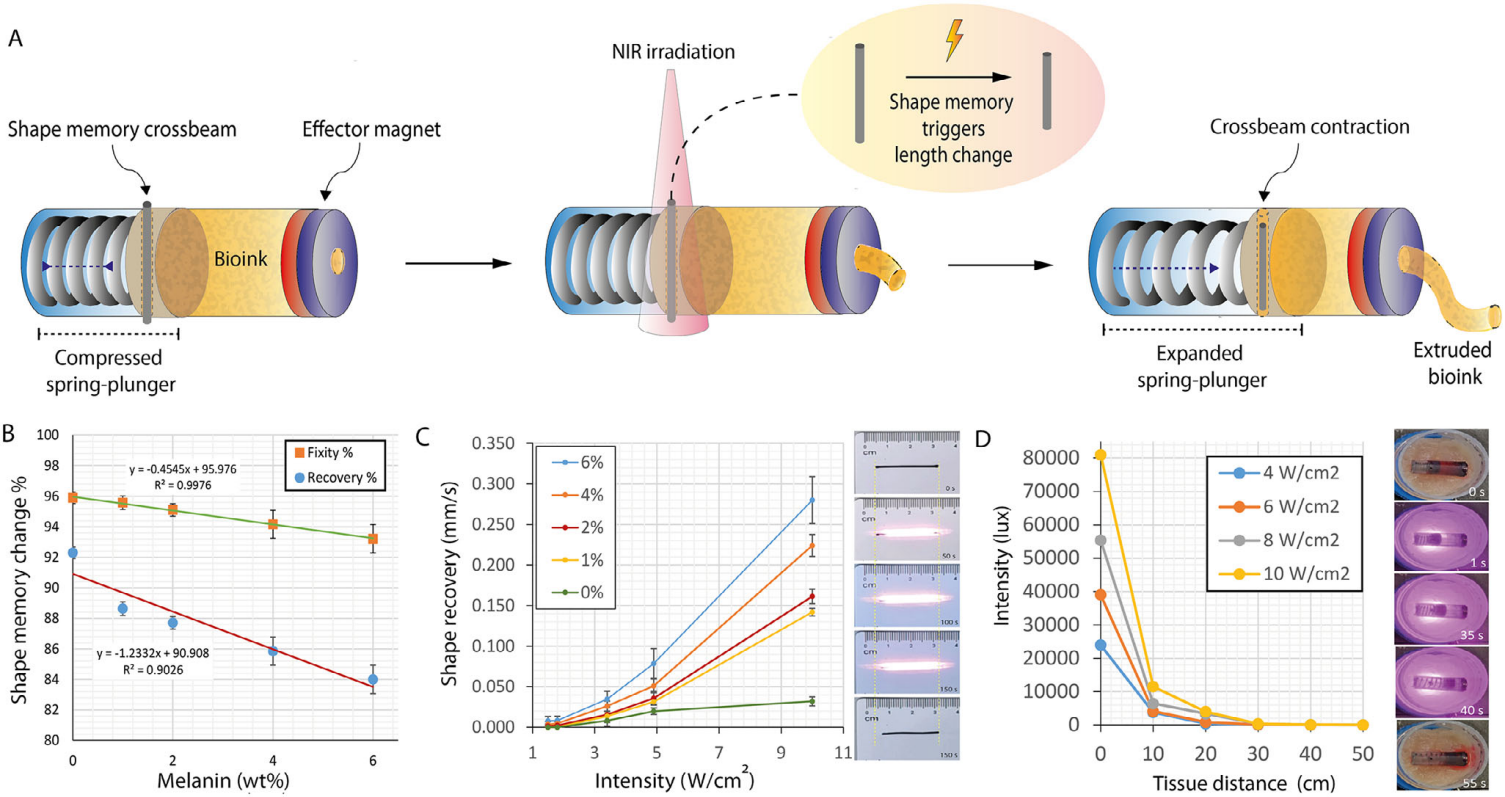
NIR‐induced bioink extrusion mechanism and shape memory characterization. A) Schematic of the NIR‐triggered bioink extrusion process using a spring‐loaded plunger, bioink reservoir, and ring magnet as an ejector orifice. The stopper, made of an NIR‐responsive shape memory crossbeam, contracts upon irradiation, releasing the plunger and ejecting bioink. B) Shape fixity and recovery of PLA‐Melanin rods at different melanin concentrations. C) Shape recovery speed versus laser intensity with representative images. D) Laser intensity at various ex vivo tissue depths and a sequence showing bioink extrusion through 7.5 mm of tissue.

To achieve ingestible‐scale bioprinting, MEDS relocates all actuation and control systems extracorporeally, utilizing NIR triggering and magnetic guidance for tetherless operation within the gastric environment. This external control paradigm is realized through a novel design where a milli‐scale biocompatible resin barrel fitted with a compressed spring‐loaded plunger, which expands to its free length when triggered via an extracorporeal NIR source. Here, the force required for bioink extrusion is provided by a compressed stainless‐steel spring (Figure S1A, Supporting Information), while the NIR‐triggered ejection is mediated by a shape memory polymer‐based stopper mechanism. This stopper mechanism holds the spring‐loaded plunger at shut‐length via a shape memory crossbeam that passes through a hole in the plunger and the barrel. The crossbeam reduces in length upon NIR exposure and facilitates the spring expansion to its free length, consequently forcing the bioink in the reservoir between the plunger and EM, as shown in **Figure** **2**A. The barrel was fabricated using stereolithography and houses a spring‐loaded plunger mechanism secured at the bottom. A gold‐coated neodymium‐iron–boron (NdFeB) ring magnet was affixed at the top, serving dual functions: magnetic steering via external actuation and acting as the bioink nozzle through its central aperture. The gold coating ensures biocompatibility, while the surrounding resin barrel encloses internal components. Designed for post‐deployment retrieval, the device maintains short intracorporeal residence time, reducing potential adverse interactions during in‐vivo deployment and recovery.

To provide robust mechanical release of the spring, the stopper beam must have sufficient fixity to hold the spring while providing sufficient shape recovery to release the spring upon triggering. Hence, we have developed NIR‐shape memory beams through melt‐extruding PLA and melanin and then investigated their properties. Melanin was used here for its photothermal conversion property and provide the thermal energy for the shape memory trigger.^[^ ^39^ ^]^ Previously, the negative impact of a guest material on the shape memory properties of the host polymer has been reported.^[^ ^41^, ^42^ ^]^ From the data in Figure 2B, we observe a similar behavior, where the fixity % decreases with the increase in melanin content. Nevertheless, greater than 90% fixity and 80% recovery were observed for all the melanin concentrations used herein. In addition, we tested the shape recovery under 808 nm illumination and observed an increase in recovery speed proportional to the applied intensity as shown in Figure 2C. Additionally, we confirmed its function as a crossbeam stopper in the spring‐loaded plunger assembly by testing it through 7.5 mm of ex vivo muscle tissue using a 350 J cm^−^^2^ fluence rate (10 W cm^−^^2^ for 35 s), as shown in Figure 2D, consistent with clinical studies on photodynamic therapy.^[^ ^43^, ^44^ ^]^ Therefore, we have developed and demonstrated a suitable trigger mechanism for bioink extrusion triggerable from outside the body.

### 2.2 Surface‐Mediated Positioning and Magnetically Modulated Stick‐Slip (MMSS) Dynamics

**Figure 3:**
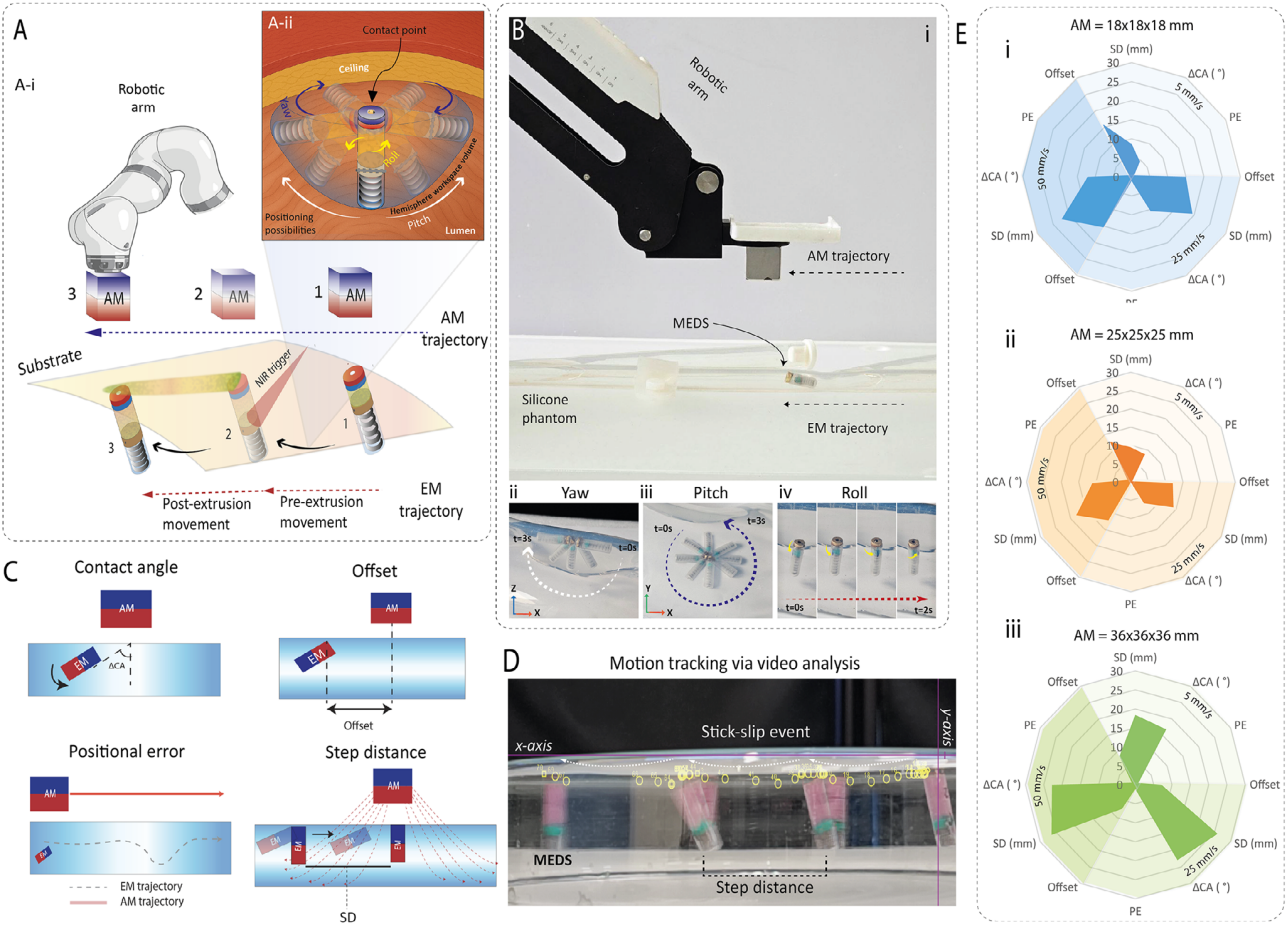
Pre‐extrusion actuation: Characterization, Optimization, and Performance Analysis. A‐i) Schematic distinguishing pre‐ and post‐extrusion actuation based on the bioink interface between the EM and print substrate. A‐ii) Ceiling‐constrained positioning showing pitch–yaw sweep around a contact point, illustrating working volume possibilities. B‐i) Experimental AM–EM setup with silicone phantom showing trajectory alignment. B‐ii) Yaw rotation of the EM under AM control. B‐iii) Pitch rotation of the EM demonstrating hemispherical sweep. B‐iv) Roll rotation enabling 360° axial freedom. C) Illustration of responses – offset, step distance (SD), contact angle difference (ΔCA), and positional error (PE). D) Experimental AM–EM setup on a glass substrate, with motion tracking via video analysis, showing stick–slip motion. E) Radar charts analysing AM cube dimensions (18 mm, 25 mm, 36 mm) across velocities (5–50 mm s^−1^), highlighting optimal EM manoeuvrability for 18 and 25 mm cubes.

Having established the extrusion mechanism, we next characterized the magnetic actuation system required for positioning and printing. To enable untethered in‐situ printing, we describe and optimize the AM‐EM interaction and EM behavior on the lumen ceiling (directly on the tissue surface, pre‐extrusion or with a lubrication layer of bioink, post‐extrusion). Typically, accurate magnetic positioning is achieved in conjunction with other non‐magnetic forces or with complex multi‐axis magnetic fields, as in previous microbot implementations.^[^ ^45^, ^46^ ^]^ Our AM‐EM configuration, however, has a surface‐mediated, ceiling‐constrained configuration and it navigates through a unique “step‐slide” gait at the lumen ceiling. Hence a novel way to enable controlled stick‐slip movement at the lumen ceiling was modeled. First, the actuator magnet (AM) moves and increases its *x*‐axis separation from the EM (the “step”). This is followed by a swift “catch‐up” step (the “slide”) by the EM to reduce the separation (**Figure** **3**D; Note S1, Supporting Information). This is explained by the fluctuating interplay between the frequently changing horizontal (Fx) and vertical (Fy) magnetic force components, leading to periodic stick‐slip events. The presence of this dynamic force component, combined with the traditional stick‐slip in our surface‐mediated positioning strategy, was termed magnetically modulated stick‐slip (MMSS). These motion dynamics can be modeled by balancing magnetic and friction forces:

(1) $$\frac{md^{2}x}{dt^{2}}=\frac{k\left(x_{1}-x_{2}\right)}{r^{4}}-\mu_{k}N\left(\frac{dx_{2}}{dt}\right)$$

where ***x*_1_**, ***x*_2_**, and ***h*** define the magnet positions and separations (Supplementary text). After extrusion, the EM is lubricated by the bioink interface and reduces both the static (µ_s_) and kinetic friction (µ_k_) coefficients. This bioink interface also introduces fluid‐mediated damping for absorbing oscillations and stabilizes the microdevice navigation, which is described as:

(2) $$\frac{md^{2}x}{dt^{2}}+C\;\frac{dx_{2}}{dt}=\frac{k\left(x_{1}-x_{2}\right)}{r^{4}}-\mu_{k}N\left(\frac{dx_{2}}{dt}\right)$$

where *C* represents the damping coefficient from the bioink.

These derivations show the negative effect on the positioning accuracy arising from MMSS. This may be alleviated by employing extruded bioink to lubricate the interface between the printer and the tissue. Thus, a strategy for accurate pattern formation is suggested, wherein the bioprinter should be guided to a target location, and the post‐extrusion pattern formation can then be achieved by spreading the extruded bioink. Therefore, our experimental efforts focused on optimizing parameters such as AM geometry, velocity, and magnetic coupling distance to minimize stick‐slip‐induced errors and to achieve reliable and accurate navigation. Additionally, this mathematical model served as an interpretive tool for our experimental observations.

### 2.3 Magnetic Positioning Optimization

Given the occurrence of MMSS, we aim to reduce the spatiotemporal discrepancies between the AM and EM trajectories sufficiently to achieve the target accuracy needed for ulcer sealing. Hence, we focused on improving positioning accuracy via optimizing experimental factors such as AM dimensions and velocity, and observed their influence on EM trajectory. With our AM‐EM setup, two distinct operational regimes: pre‐ and post‐extrusion positioning, which differed based on the presence or absence of bioink at the interface between the EM and substrate (Figure 3A‐i,B‐i). We ultimately selected a single AM‐EM Configuration that provided sufficient positioning accuracy in both regimes of operation.

#### 2.3.1 Pre‐extrusion Positioning

Acknowledging the transient positioning errors introduced by MMSS, we have studied the effect of various factors on the positioning accuracy of the EM through responses (Figure 3C) such as positional error (PE) – the z‐test value between the coordinates distribution of AM and EM path; contact angle (ΔCA); and step distance (SD) due to MMSS for various factors such as AM dimensions (18–36 mm) and velocities (5–50 mm s^−1^), through video analysis as shown in Figure 3D. Among the tested AMs, the 25 mm cube at 5 mm s^−1^ showed the best results; this was closely followed by the 18 mm cube at the same speed, while the 36 mm cube performed poorly at all AM velocities (Figure 3E). Using the optimized AM‐EM settings, MEDS workspace characterization in gastric phantoms demonstrated full 6°of freedom control, achieved by varying AM orientations to generate different magnetization directions. This enabled complete 360° rotational freedom (yaw, pitch, roll). It is also capable of unlimited rotations, arbitrary orientations, full inversion, and independent positioning without path constraints, capabilities that are impossible with tethered systems. Tether‐induced issues which limit tool freedom, like constraints from the sheath or drive shaft, torsional buildup, restricted orientations due to bending damage etc., are absent in our MEDS system^[^ ^47^, ^48^, ^49^, ^50^ ^]^ (Figure 3A‐ii,B‐ii–iv). It also achieves a hemispherical working volume of 5747 mm^3^ with angular resolutions of 3.23° ± 0.59° (pitch), 3.67° ± 0.73° (yaw), and 12.33° ± 1.38° (roll) under ideal conditions, providing distinct positioning advantages over contemporary tethered systems (Table S1, Supporting Information).

In tethered tools, mechanical constraints from the sheath, driveshaft, and optical fibers physically restrict free inversion or continuous rotation, while cable twist buildup prevents unlimited motion. Extreme bending or deflections risk shaft damage, enforcing narrow angular safety range, and stiffness factors in flexible shafts or rotary joint designs limit bending angles and prevent 3D tumbling or tip flips. Together, these tether‐induced constraints, torsional buildup, restricted orientations, bending damage, and limited operational envelope, severely curtail spatial freedom compared to our tetherless MEDS approach.

#### 2.3.2 Post‐Extrusion Positioning

**Figure 4:**
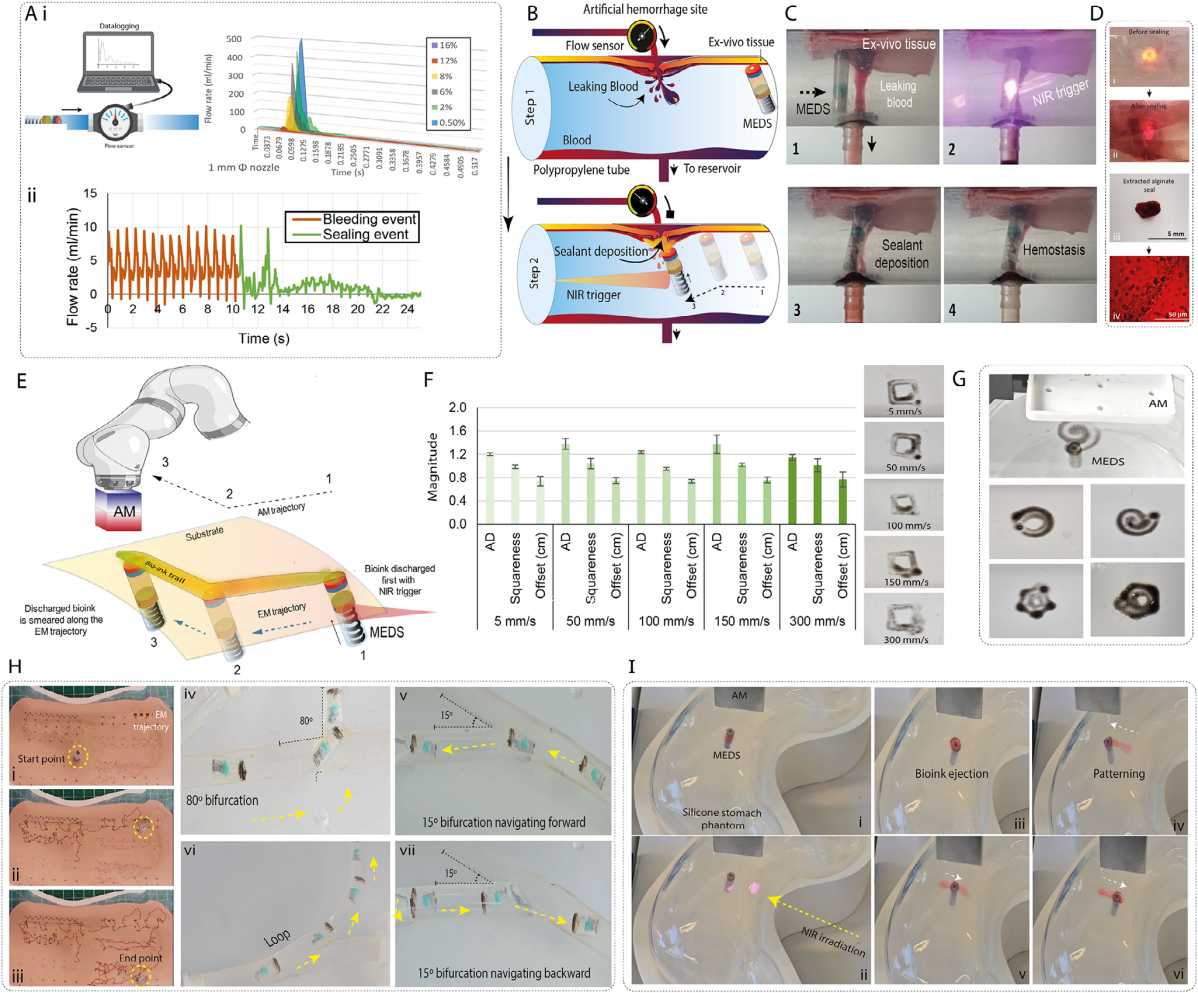
Flow Characterization, Hemostatic Sealing, and in vitro actuation studies. A) i) Flow rate characterization setup containing a flow sensor, and datalogging system connected to MEDS. ii) Blood flow rate before and after sealing. B) Illustration of hemostatic sealing in an ex vivo hemorrhage model (2 mm incision, 5 mL min^−1^ flow). C) Sequential images depicting the sealing process. D) i, ii) *Ex vivo* perforation before and after alginate sealing. iii, iv) Macroscopic and microscopic images of the extracted alginate seal. E) Dispense and Daub (DnD) strategy for bioink patterning. F) Quantitative analysis of printed squares (area deviation and squareness) at varying speeds (18 mm AM). G) Post‐extrusion printing of various shapes. H) Pre‐extrusion positioning in a waypoint navigation course and positioning in silicone phantoms with bifurcations and loops. I) Pre‐ and post‐extrusion positioning sequence. I) i, ii) Navigation and NIR‐trigger application. I) iii) Bioink extrusion. I) iv–vi) EM‐actuated patterning into a linear construct.

We used glass as a preliminary printing substrate to enable video analysis‐based measurements and an alginate bioink for preliminary demonstrations. The bioink payload was synthesized mixing DMEM culture medium with various concentrations of sodium alginate (0.5–16 wt%). Alginate was selected here due to its stability, tunable viscosity, biocompatibility, mucoadhesiveness, in vivo degradability, and non‐immunogenicity.^[^ ^51^, ^52^, ^53^, ^54^, ^55^, ^56^ ^]^ We then investigated the flow profile of the extrusion event with various nozzle diameters (1.0–1.8 mm, Figure S1B,C,D, Supporting Information) and bioink viscosities (0.1–250 Pa.s, Figure S1E, Supporting Information). The results showed that neither varying the viscosities nor the nozzle diameters imposed significant pneumatic dampening on the spring, as shown in **Figure** **4**A. This quick‐pulse bioink unloading is considered undesirable for conventional bioprinting, in which the print path and extrusion are time‐synchronized, but we found it useful for arresting hemorrhages. Alginate is an established hemostatic agent, due to its ability for blood contact induced‐gelation,^[^ ^57^ ^]^ platelet activation ^[^ ^58^ ^]^ and calcium ion exchange,^[^ ^59^ ^]^ making it an appropriate material for this application. We demonstrated hemostasis using an artificial ex vivo haemorrhage platform, with a biologically‐relevant hemorrhage flow rate^[^ ^60^, ^61^ ^]^ (Figure 4B). By magnetically directing MEDS to the haemorrhage site, applying pressure through AM‐EM distance reduction (Figure S1, Supporting Information), and dispensing alginate, we were able to arrest blood flow, as shown in Figure 4C,A‐ii and Movie S2 (Supporting Information). A robust, leak‐proof seal of the hemorrhage was achieved post‐dispensing, which we confirmed visually and by flow sensor data (Figure 4C,D).

The lack of sustained extrusion led us to adopt a simplified yet novel bioprinting approach termed “dispense‐and‐daub” (DnD). Here, bioink is extruded at the target site and later shaped by controlling the EM trajectory with the AM, overspreading the ink (Figure 4E). This decouples the need for synchronization between the AM trajectory, NIR triggering, and extrusion. The lubricating bioink interface also largely eliminated stick‐slip‐induced stepping. While not a layered, time‐synchronized process, DnD is suited for constrained, in‐situ environments and represents a 2.5D printing strategy much like screen printing^[^ ^62^ ^]^ or inkjet based bioprinting.^[^ ^63^ ^]^ Alternatively, we also tested a friction‐based dampening strategy for sustained spring release and extrusion. We used a barrel with an internal diameter gradient to subject the plunger to a higher frictional force at the bottom and a lower frictional force as it moved upward, resulting in a sustained release for up to 15 s (Figure S1F–H, Supporting Information). However, we did not use this in the present study since DnD yielded good results without synchronization issues.

To optimize DnD, we investigated three variables affecting print performance: daub repetitions, AM dimensions and AM velocity. Key responses measured were area deviation (AD), aspect ratio, and offset between AM position and EM contact point. The 18 mm AM cube provided superior print fidelity at 5–50 mm s^−1^ (Figure 4F), and the offset remained largely unchanged between pre‐ and post‐extrusion states. We found that the required coupling distance for maneuvering EM was 56 ± 1.5 mm (Figure S3A–D, Supporting Information), and that five daub repetitions were optimal for an ideal square (Figure S3C, Supporting Information). The 18 mm AM at 5–50 mm s^−1^ was optimal for simplifying the integration of pre‐ and post‐extrusion operation. Further, 3 wt.% alginate provided optimal spreading and shape retention while maintaining biocompatibility (Figure S2A‐ii, Supporting Information). Using this configuration, we demonstrated successful patterning circles, spirals, stellates, and polygons (Figure 4G).

With these optimized parameters, we could now validate the positioning accuracy of MEDS using a waypoint navigation test on an ex vivo surface patterned with a dot grid of 0.7 mm target dots separated by 7 mm distance (Figure 4H i‐iii) and navigate it through narrow lumen architectures (Figure 4Hiv–vii; Figure S7, Supporting Information). The mucin‐lubricated ex vivo surfaces reduced pre‐extrusion stick‐slip to a great extent, with only intermittent stick–slip, the EM successfully targeted 64 of 70 dots (91.4% success rate, Movie S3, Supporting Information), confirming that precise positioning is possible for practical patterning tasks even under MMSS conditions. However, positioning over dots spaced at 1 mm distance could not be reliably achieved. We further demonstrated phantom gastric printing (Figure 4I; Movie S4, Supporting Information), which involved aligning MEDS, extruding the bioink with an NIR trigger, and patterning a linear construct in a silicone stomach phantom. These alginate constructs were crosslinked post‐printing using 0.5m CaCl_2_, as described elsewhere.^[^ ^64^ ^]^ In vivo, we could achieve crosslinking via ingestion of a GRAS‐labeled CaCl_2_ solution.^[^ ^65^, ^66^ ^]^ Such ingestion protocols are routinely used in clinical workflows like Barium for CT or water for ultrasound,^[^ ^67^, ^68^ ^]^ or a dual‐device system could dispense bioink and crosslinker separately. Although the system currently uses alginate as a demonstration material it can also accommodate a range of biomaterials with suitable rheological properties. These include shear‐thinning for smooth extrusion, rapid viscosity recovery to preserve construct shape, and an appropriate viscosity window that enables adjacent printlines to coalesce while preventing uncontrolled flow. Materials such as gelatin, fibroin, and collagen can be tuned to meet these requirements through concentration adjustment, viscosity modifiers or crosslinking strategies.

### 2.4 Print Topography Optimization

**Figure 5:**
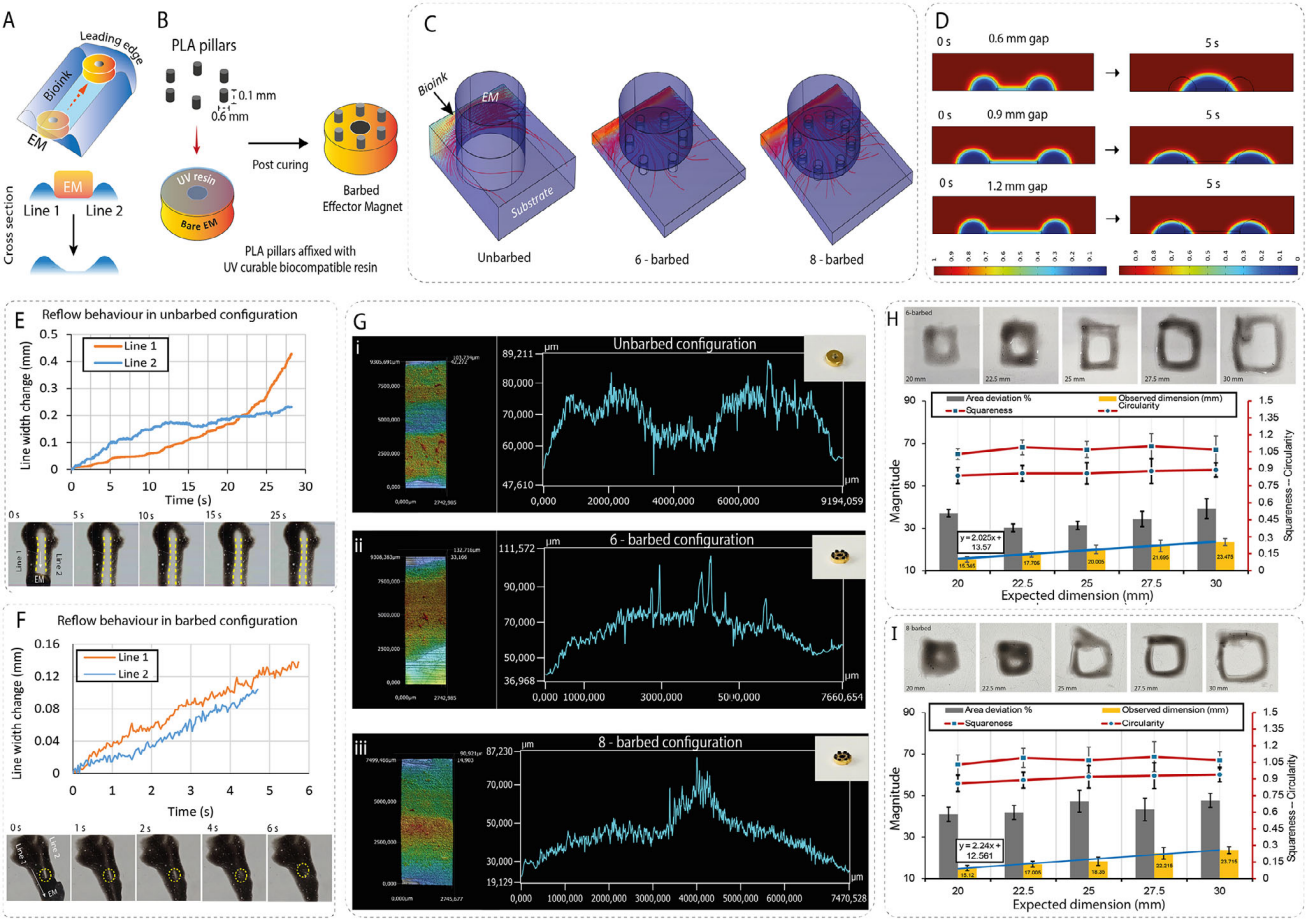
Illustration of unbarbed prints, construction of barbed EMs, and in‐silico optimization. A) Illustration showing print line displacement due to EM trajectory. B) Fabrication of the barbed‐ejectors or EMs. C) CFD simulations in COMSOL showing streamline distributions for different barb configurations. D) 2D simulations analyzing threshold distances between barbs for complete coalescence of separated bioink printlines. E,F) Video analysis of reflow behavior in unbarbed and barbed printlines. G) Height map and cross‐sectional profile of coalesced printlines from laser confocal microscopy in unbarbed, 6‐barbed, and 8‐barbed configurations. H,I) Quantitative analysis of printed squares, showing differences in print characteristics between 6‐barbed and 8‐barbed ejector configuration.

While the optimized DnD parameters enabled successful pattern formation, analysis of the printed constructs revealed opportunities for further enhancement of print line quality. Specifically, the DnD process leads to uneven bioink distribution across the print line, resulting in a double peak topography as indicated in **Figure** **5**A. We attribute this print topography to bioink displacement along the ejector surface during ink spreading, where flowlines may diverge to the EM perimeter to cause the formation of ridged ink patterns. By incorporating a barbed‐ejector (EM) design (Figure 5B), we could reduce the distance between the adjacent lines, allowing the ink lines to approach each other, facilitating total coalescence. We first validated this using CFD simulations (Figure 5C), where 6‐barb and 8‐barb configurations were compared; the latter showed better bioink homogeneity due to a dispersed streamline distribution pattern. Further, we used a Phase field in Fluids module to tune the distance between barbs for coalescence; we found the ideal spacing to be 0.6 mm, as seen in Figure 5D.

Subsequently, we experimentally verified the simulation results by constructing barbed‐ejectors using ring magnets affixed with PLA pillars of 0.6 mm diameter and 0.1 mm height, which were fabricated using biocompatible resin. Unbarbed configuration prints exhibited a prominent double peak ink profile under confocal microscopy, as seen in Figure 5G‐i. These displaced print lines nonetheless exhibited some reflow behavior and reduced the discontinuity partially, as seen in Figure 5E. In sharp contrast, we found that the barbed configurations produced coalesced printlines with a single central discontinuity of about 0.1 mm, which coalesced within 6 s, as indicated in Figure 5F. Compared to the unbarbed printlines, both the barbed configurations showed a well‐coalesced cross section with a central height of about 70 microns in through confocal microscopy (Figure 5G‐i–iii).

Thus, the barbed configurations enhanced the bioink distribution across the printlines. By printing test squares with these barbed EMs, we found that the barbs provided consistent dimension offsets between the desired and printed squares due to the barbs blocking the original trajectory, as seen in Figure 5H,I. Importantly, the 6‐barbed configuration was better than the 8‐barbed design for square printing in terms of circularity and area deviation, and thus is preferred for controlled bioink printing in the body.

### 2.5 Ex‐vivo Ulcer Coatings and Printing With Cell‐Laden Bioinks

**Figure 6:**
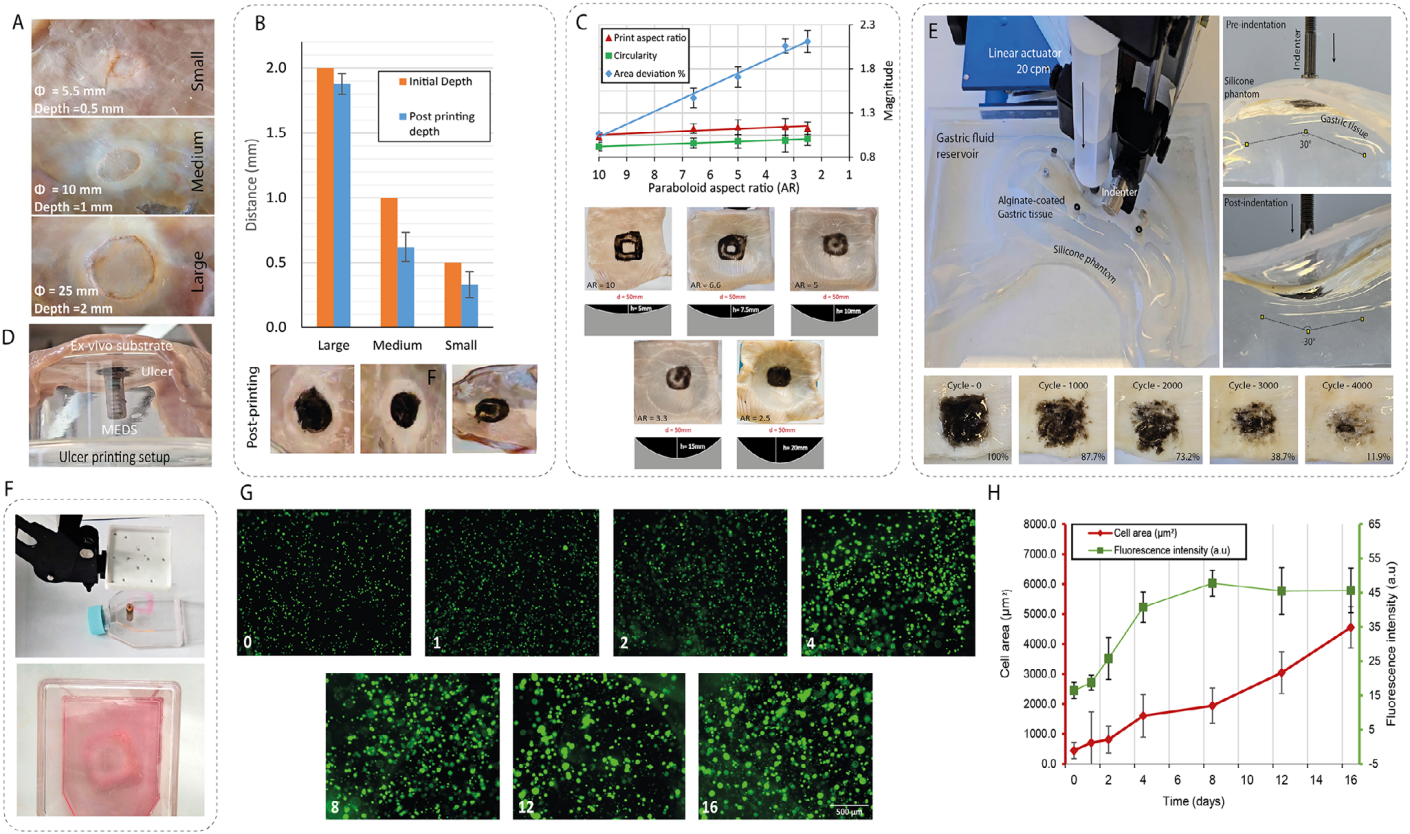
Ex‐vivo printing – Characterization and performance analysis. A,D) Images show ulcers of increasing size and depth created by tissue excision, alongside the ulcer printing setup. B) A plot illustrates ulcer depth reduction post‐printing, with experimental images of spirals printed over ulcers. C) Conformal printing feasibility is assessed using ex vivo tissue placed over concave paraboloid structures, with plots detailing area deviation, circularity, and aspect ratio. E) Fatigue testing involves alginate‐coated gastric tissue subjected to cyclic bending (±30°, 20 cycles/min, 2.2 N) in simulated gastric fluid, showing adhesion loss over 4000 cycles. F) Images show the EM‐AM actuation setup in a T‐25 flask and crosslinked square prints in DMEM. G,H) Fluorescence microscopy tracks cell area and intensity over time, with results in a scatter plot.

After testing MEDS in silicone phantoms, we assessed its performance on ex vivo ulcer constructs. We observed similar print fidelity to glass substrates during preliminary tests on ex vivo gastric tissue (Figures S3,S4 and Movie S5, Supporting Information). To evaluate ulcer printing feasibility, ex vivo ulcers of varying dimensions (small: *Φ* = 5.5 mm, depth = 0.5 mm; medium: *Φ* = 10 mm, depth = 1 mm; large: *Φ* = 25 mm, depth = 2 mm) were created (51,52) on gastric tissues (Figure 6A,D; Movie S6, Supporting Information). The device closely followed the intended spiral trajectory on small ulcers but exhibited slightly reduced spatial accuracy due to the ulcer bevel; However, despite the effect of the bevel, the ex vivo ulcer was successfully sealed. For medium and large ulcers, the device circled entirely within the ulcer perimeter, unable to climb sidewalls. Interestingly, this behavior enhanced ulcer sealing by confining bioink within the targeted region, assisted by capillary action from sub‐mucosal tissue striations, resulting in improved coverage and reduced ulcer depth (Figure 6B). To mimic realistic in vivo geometry, we printed seals on ex vivo gastric tissue over paraboloid‐concave substrates (Figure 6C). Print aspect ratio, circularity, and area deviation increased with substrate curvature, yet concave structures with aspect ratios below 6.6 minimally impacted print fidelity, suggesting suitability for application in the upper GI tract.^[^ ^69^ ^]^ Durability tests in a mechanically‐perturbed, gastric simulator containing simulated gastric fluid confirmed stability of printed alginate constructs, with ≈73% retention post‐patterning, suggesting the applicability of this technique for haemorrhage sealing or transient ulcer dressings (Figure 6E; Figure S5A,B, Supporting Information) where short residence times have shown to accelerate healing.^[^ ^70^, ^71^, ^72^ ^]^ Further, micromechanical effects on ex vivo tissues from repeated EM sliding contact were assessed with confocal microscopy, which showed only progressive surface smoothing consistent with microstructural compression and smoothing. No evidence of surface damage was observed (Figure S5C,D, Supporting Information).

Lastly, we evaluated cell‐laden bioink containing human gastric fibroblasts (1 million cells/ml), which maintained structural integrity over 16 days with increased cellular fluorescence intensity (Figure 6F–H). While initially lacking typical spindle‐shaped morphology due to insufficient adhesion cues, cells released following bioink degradation rapidly proliferated, recovering their characteristic morphology (Figure S4, Supporting Information).

### 2.6 In‐vivo Deployment Feasibility

Building on the successful ex‐vivo demonstrations, we conducted preliminary in‐vivo studies in a rabbit model to validate the complete workflow from ingestion to retrieval in a physiologically relevant model. The complete workflow of swallowing, steering, activation, patterning, and subsequent retrieval was demonstrated.

**Figure 7:**
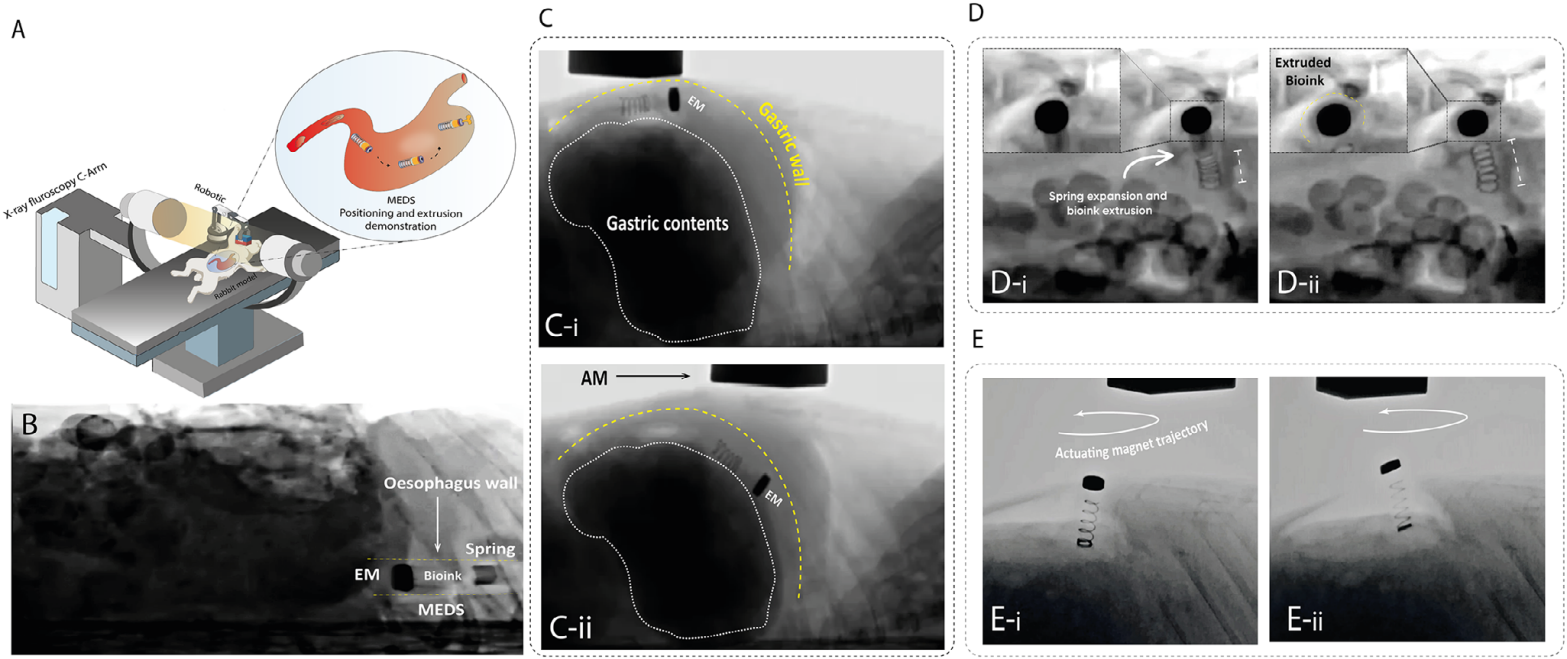
In vivo deployment of MEDS with X‐ray fluoroscopy. A) Deployment and visualization setup. B) Fluoroscopy‐assisted oesophageal positioning enabled by the radiopacity of the EM, iohexol‐blended bioink, and spring components C‐i, ii) Fluoroscopy‐assisted gastric positioning, overcoming challenges posed by gastric contents while achieving successful navigation. D‐i, ii) Fluoroscopic visualization of the bioink extrusion event. E‐ i, ii) Fluoroscopy‐guided patterning of the extruded bioink.

To assess the feasibility of device navigation and actuation in a live anatomical setting, we conducted preliminary in vivo tests in a rabbit model (**Figure** **7**A; Movie S7, Supporting Information). These trials were explicitly intended to evaluate magnetically‐guided ingestion, steering, extrusion, and structured bioink deposition. Real‐time X‐ray fluoroscopy enabled visualization of the radiopaque components of the device (Figure 7B). We successfully demonstrated controlled bioink extrusion and patterned deposition on the stomach ceiling (Figure 7D‐i,ii, E‐i,ii). Active forward and reverse navigation were validated under operator control across key gastric landmarks, including the pylorus, fundus, greater curvature, and oesophagus, despite variable gastric contents and constrained volume (Figure 7C). The device reproducibly followed predefined actuator trajectories in two separate trials, confirming reliability of the AM‐EM control strategy. NIR‐triggered extrusion was performed through intact tissue using an extracorporeal source with iohexol included in bioink for radiopacity. The device was then retrieved via the same oral route using magnetic steering, avoiding passive excretion and enabling potential repositioning or redeployment. We note these in vivo experiments were not designed to evaluate therapeutic efficacy. No healing, biological integration, or treatment outcomes were assessed. Future studies using disease models will be required to assess clinical impact and therapeutic potential.

## 3 Discussion

We present MEDS, a novel tetherless bioink deposition system that enables bioink patterning through a novel tetherless bioink deposition system that enables patterned deposition through a medication‐like platform, in contrast to the procedure‐based interventions required by previous systems. Through systematic progression from device design and magnetic actuation modeling to experimental optimization, ex vivo validation, and in vivo feasibility demonstration, we establish a foundation for future in vivo bioprinting. Using magnet‐NIR hybrid actuation in place of onboard electronics, MEDS enables precise endoluminal positioning and printing via an effector magnet (EM) guided by an actuator magnet (AM) on a robotic arm to navigate complex GI geometries, deposit bioink, and enable oral retrieval. The system surpasses existing in‐situ printers in miniaturization, non‐invasiveness, and tetherless operation, facilitating access to anatomically challenging regions while minimizing procedural trauma (Table S1, Supporting Information).

The AM‐EM configuration allowed ceiling‐constrained, surface‐mediated navigation to suppress magnetically induced stick‐slip. Optimal results were achieved using an 18 mm cubical AM actuated at 5–50 mm s^−1^, maintaining a 55 mm coupling distance. For lower‐fidelity needs, the 35 mm AM maybe used to extend the range to ≈85 mm. We also introduce a 2.5D bioprinting approach, termed dispense‐and‐daub (DnD), which, like other 2.5D methods, forms patterns, but does so by shaping deposited bioink via EM trajectory, without requiring synchronized extrusion and printhead motion. Further, barbed ejectors were designed and optimized to improve coalescence and print uniformity. The device sealed artificial hemorrhages and successfully patterned ulcer dressings in ex vivo tissue using a fully tetherless workflow. Printed constructs retained 73% of alginate mass under gastric simulation without delamination. Alginate‐based cell‐laden constructs remained viable for over 16 days, suggesting potential as micro‐bioreactors that release growth factors and recruit progenitor cells for wound healing.^[^ ^73^ ^]^ Unlike monocultures, where rapid proliferation leads to nutrient depletion and apoptosis,^[^ ^74^ ^]^ alginate's slower proliferation supports long‐term viability (up to 150 days).^[^ ^73^ ^]^ This could enable sustained delivery of cells, gradually released via calcium crosslink degradation and proliferating at the wound site. Finally, in vivo rabbit studies demonstrated the feasibility of fluoroscopy‐guided positioning, extrusion, and patterning. These results indicate physiological applicability and establish its foundational role in future endoluminal bioprinting applications.

## 4 Future Work

Expanding the AM‐EM working distance using stronger magnets or Halbach arrays, alongside parameter tuning, will be crucial to gain access to deeper organs. Manual control may also be eliminated via algorithmic path planning, closed‐loop sensor feedback, and compensate for peristaltic disruptions. The lifespan of current scaffolds is limited by gut mucosal turnover and peristaltic forces.^[^ ^75^ ^]^ Future work should focus either on leveraging short‐residence dressings, since healing outcomes are not strictly tied to scaffold duration ^[^ ^70^, ^71^, ^72^ ^]^ or on strategies to enhance construct retention^[^ ^54^ ^]^. To simplify the workflow, future iterations may also employ crosslinking‐free strategies using silk fibroin,^[^ ^76^ ^]^ thermosensitive bioinks,^[^ ^77^ ^]^ native enzyme‐activated crosslinking^[^ ^78^ ^]^ or body temperature‐triggered enzymatic crosslinking.^[^ ^79^ ^]^ While pristine alginate was used as a demonstration material in our system and supports cell viability, it lacks adhesion motifs required for effective cellular dispersion ^[^ ^53^, ^80^ ^]^ as observed in our experiments and supported by literature.^[^ ^73^, ^81^ ^]^ Future work will focus on the addition of adhesion peptides like RGD or using other bioinks that improve cellular morphology.^[^ ^82^ ^]^

While this study demonstrates real‐time navigation, actuation, and deposition under fluoroscopy, it does not validate therapeutic efficacy. Future in vivo studies using ulcer or hemorrhage models are needed to quantify healing metrics over time, supported by histology. In summary, our tetherless ingestible bioprinter demonstrates the ability to deliver and pattern bioinks within the GI tract without invasive procedures or restrictive tethers. Despite the limitations, this foundational work represents a significant step forward compared to the current bolus drug release ingestibles.^[^ ^83^.^]^ In summary, by uniting the worlds of ingestibles and bioprinting, we realize the vision of a pill that prints, which opens paths to future ingestibles that do not just deliver molecules, but actively construct tissue in the very spaces where disease took hold.

## 5 Experimental Section

### MEDS Design and Fabrication

The shape memory crossbeam was prepared using polylactic acid (PLA), which has inherent shape memory properties.^[^ ^84^ ^]^ The PLA was rendered NIR‐triggerable by compositing it with melanin,^[^ ^85^ ^]^ which was chosen for its excellent biocompatibility and NIR photothermal conversion properties.^[^ ^39^ ^]^ The effect of various concentrations of melanin on the shape fixity and recovery properties of PLA with melt extruded shape memory beams and standard bend testing characterization was studied.^[^ ^86^ ^]^ MEDS featured a milli‐scale barrel that incorporated an NIR‐dependent spring‐loaded plunger mechanism for extruding bioink. A barrel of 14 mm height, 6 mm outer diameter, and 1 mm wall thickness was stereolithography (SLA) printed with Phrozen Mini 8K printer, using a biocompatible photopolymer resin sourced from Liqcreate. A stainless‐steel spring (h = 14 mm, *φ* = 4 mm) attached to a plunger bit from an insulin syringe was used to create the spring‐loaded plunger mechanism, which was positioned at the bottom of the tube, while a gold‐coated NdFeB (Neodymium iron boron) ring magnet (outer *φ* = 5 mm, inner *φ* = 1 mm), acting as the effector magnet (EM)., was press fitted on the top of the tube. These NdFeB magnets were procured from Webcraft AG, Switzerland, and their inner diameters were varied to assess the pneumatic dampening effect on the spring. Approximately 200 µL of bioink was held in the space between the plunger and EM, this space serving as the reservoir. Inside the milli‐scale barrel, the plunger‐spring mechanism was compressed and held in its compressed shut‐length state by an NIR‐shape memory crossbeam inserted through a pair of holes traversing the barrel diameter. The crossbeam undergoes structural deformation upon exposure to NIR radiation, allowing the spring to freely extrude the bioink in the barrel reservoir. The resulting final device has specifications of *Φ* = 6 mm, length =  14 mm length and weight ≈ 28 mg.

### Characterization of Shape Memory Properties

The shape memory characteristics of the stopper crossbeam was evaluated using two important parameters – shape fixity and shape recovery. Samples were chosen that were 0.3 mm thick and 5 mm long and were prepared by melt extrusion using a 3devo Maker ONE filament maker. The evaluation was carried out through a standard bending test. Here, the sample was first bent from θi to θp (5° to 175°) after incubation at 115 °C for 2 min above T*trig* and was held in this form. In the second step, the bent posture was maintained with the help of an external force provided by a clip for 2 h at 7 °C. Next, the external force was removed in order to record the fixed angle θf. Finally, to test its recovery characteristics, the sample was heated to T*trig* (45 °C) and documented the recovered angle θr. The fixity ratio R*f* and the recovery ratio R*r* were calculated using the formulae $R_{f}=\left(\frac{\theta_{f}-\theta_{i}}{\theta_{P}-\theta_{i}}\right)\;\times 100$ and $R_{r}=\left(\frac{\theta_{f}-\theta_{r}}{\theta_{f}-\theta_{i}}\right)\;\times 100$ respectively.^[^ ^87^, ^88^ ^]^ Our evaluation of NIR‐triggered shape memory was confirmed by using the same beams heat programmed to a length of 10 mm from 7.5 mm, and assessing their recovery to native length through exposure to an overhead NIR laser source procured from Roithner Lasertechnik GmbH, Austria. For checking the NIR‐triggered bioink extrusion through tissues, MEDS was placed over 7.5 mm of ex vivo chicken breast and irradiated with the laser source from below.

### Flow Characterization Setup

Flow measurements were performed using an SLF3S‐4000B sensor evaluation kit, which is capable of measuring up to 1 L min^−1^ flow rates. Then, this sensor was connected using a Digital interface (I2C) via a 6‐pin standard electrical connector to a laptop, and data was collected through the proprietary Sensirion RS485 software. Finally, MEDS was triggered and observed the flow stemming from the extrusion event using the software serial monitor.

### EM‐AM Actuator System

To achieve positioning control of MEDS via magnetic guidance, cube‐shaped N52 grade NdFeB magnets of varying dimensions procured from KJ Magnetics, USA, onto a robotic arm (Dobot Magician MG‐4R005‐02E) were mounted. The minimum coupling distance was assessed by adjusting the z‐distance between the AM and the EM. Post‐coupling, predefined print paths were executed using the control software, DOBOT Studio. For print paths involving complex shapes, vector files was generated on Inskscape software and uploaded these files into the writing menu of DOBOT Studio. The pitch and yaw demonstrations were carried out with the AM mounted on UR5e cobot. A video was also used to record EM responses, such as EM velocity, step distance etc., and then analyzed the video using Tracker from Open‐Source Physics, through a combination of manual keyframing and autotracking. The contact angle difference between the EM and substrate was calculated using the formula Δ*CA*  = 90^0^  − θ_*EM*_. The *z*‐score for the positional error was calculated between the distributions of the y‐coordinates between AM trajectory and EM trajectory using the formula $z=\left({\bar{x}}^{1}-{\bar{x}}^{2}\right)/\surd \left(\left(\sigma_{1}^{2}\right)/n_{1}\;\left(+\sigma_{2}^{2}\right)/n_{2}\right)$ where, ${\bar{x}}_{1}$ is the mean of the first sample (AM). ${\bar{x}}_{2}$ is the mean of the second sample (EM), $\sigma_{1}^{2}$ is the variance of the first sample (AM). $\sigma_{2}^{2}$ is the variance of the second sample (EM), n_1_ and n_2_ are the sample sizes of AM and EM, respectively.

### Bioink Formulation and Printing

A bioink was synthesized by mixing various concentrations of sodium alginate (≈26 000 Da, from *Macrocystis pyrifera*, Sigma Aldrich), ranging from 0.5 to 16 wt.%, with DMEM tissue culture medium. The varying alginate concentrations provided the desired rheological properties to assess the pneumatic dampening potential. After printing them on glass substrates, the alginate formulations were crosslinked with 0.5m CaCl_2_ solution. It was also blended with a black dye compound (Trawosa Lebensmittelfarbstoff) to enhance the contrast of the prints wherever necessary. The area deviation was calculated when printing a 20 mm square by using the formula AD = A_ds_–A_ps,_ where, A_ds_ is the desired square whose outer area (*a* ^2^ = 400 mm^2^) is negated from the inner area (*a* ^2 ^= 196 mm^2^) by assuming a stroke width of 6 mm (EM dimension), and A_ps_ is the printed square whose area is calculated similarly by negating the inner area from the outer area with ImageJ. The cross‐section of the prints was identified using Keyence VK‐X1000 laser confocal microscope. The preparation of cell‐laden bioinks was carried out with the same formulation by mixing Human Gastric Fibroblasts (Sciencell Research Laboratories) at 1 million cells mL^−1^ concentration, and the prints were carried out inside a T‐25 flask. Parallel control cultures of the same fibroblasts were seeded at ≈17 5000–20 0000 cells per T25 flask and maintained in DMEM medium under standard culture conditions (37 °C, 5% CO2) and monitored at 12, 24, 48, and 72 h time points to establish baseline cell growth and morphology (Figure S6A, Supporting Information). All bioinks, and MEDS components were sterilized beforehand via a combination of autoclaving, UV irradiation, and ethanol treatment. Post printing proliferation was analysed under fluorescence microscopy by Calcein‐AM staining in Echo revolve R4 microscope. The cell area was measured with Echo software, and the fluorescence intensity of the cells was measured from the green histogram values in Image J. For in vivo deployment purposes, it was blended with iohexol (150 mg mL^−1^) to help with radiopacity.

### *Ex‐vivo* Setups

To evaluate our device in ex vivo models, ex vivo gastric tissues from Hermann Fuchs oHG, Germany were purchased. In conformal print testing, a square shape of 70 mm dimension was well positioned over SLA 3D‐printed compliant paraboloid‐concave structures of various aspect ratios (AR = 10–2.5) with Liqreate Premium Flex resin. The print shape parameters were analysed with ImageJ. In the haemorrhage setup, a tissue section was pinned containing a 2 mm incision to a polypropylene tube and connected it to a peristaltic pump pumping blood at 5 mL min^−1^. The bottom of the tube contained an outlet for leaking blood and was connected to the main blood reservoir. Citrated bovine blood was procured from Rockland chemicals and recalcinated before the study.

### Durability Testing

The durability of the alginate prints was measured by placing ex vivo gastric tissue containing the alginate print in a simulated gastric environment. Peristaltic pumping of simulated gastric fluid at 150 mL min^−1^ (Simulated Gastric Fluid, Ricca Chemical, #710832) was maintained for 24 h, and the reduction in print area was measured and analysed using Image J software. The extent of bioink erosion and its implications for short‐term therapeutic efficacy were evaluated.

### In‐vivo Testing

All in vivo experiments were conducted at Labcorp Medical Device Development Center, USA, using anesthetized New Zealand White rabbits. The study included three male rabbits, each weighing between 3.4–4.0 kg and aged 40–50 weeks at the start. Male rabbits of this size were selected for their larger oesophageal and gastric dimensions, which are compatible with our device dimensions. The procedures were conducted under aseptic conditions, with animals placed on a heating pad to maintain normothermia. Sedation was achieved using acepromazine (0.25–1 mg kg^−1^, SQ), followed by anaesthesia with ketamine (35 mg kg^−1^, IM) and xylazine (5 mg kg^−1^, IM), and maintained using isoflurane gas anaesthesia (1%–5%) delivered via face mask. Vital signs were monitored with pulse oximetry, and ophthalmic ointment was applied to protect the corneas. MEDS was orally introduced and magnetically guided to the gastric fundus under real‐time X‐ray fluoroscopy. Near‐infrared‐triggered bioink extrusion was initiated once stable positioning was confirmed, followed by patterned deposition and device retrieval along the original oral path. The total procedure duration per animal was ≈30 min. Recovery of the device through the oral route after deployment through the lower oesophageal sphincter presented a challenge. However, significantly reduced sphincter tone due to isoflurane, real‐time fluoroscopy, magnetic guidance coupled with the smaller device footprint enabled reverse passage through the oesophagus. Future studies will evaluate sphincteric response and retrieval success rates in larger animal models. Animals were euthanized immediately following the procedure via intravenous pentobarbital overdose, in accordance with AVMA guidelines. The study protocol was approved by the Institutional Animal Care and Use Committee (IACUC #2661, ANS #2883) and the EPFL Animal Research Ethics Committee (AREC Application ID: AREC000043). No histological analysis was performed in this preliminary feasibility study; future work will assess inflammatory responses, mucosal integration, and long‐term safety.

### Data Analysis

Statistical Analysis – All quantitative experimental values were presented as *n* = 3, mean ± standard deviation (SD) of the mean. Computational Simulations Computational fluid dynamics (CFD) simulations were conducted using COMSOL Multiphysics to model bioink distribution during the daubing process. The simulations explored the coalescence of the bioink droplets with relation to their viscosity using the Phase field in Fluids 2 Multiphysics module. The flowline distribution of the bioink in response to various EM barbed configurations was identified using the Laminar Flow module. The results were validated against experimental data to refine the design of the MEDS's effector magnet system.

## Conflict of Interest

Authors declare financial interest in the form of a patent application.

## Author Contributions

**S.M**. contributed to conceptualization, visualization, validation, methodology, experimental investigation, data curation, wrote, reviewed, and edited the final manuscript. **V.S**. contributed to conceptualization, validation, supervision, methodology, formal analysis, data curation, and acquired resources and funding, and wrote, reviewed, and edited the final manuscript.

## Supporting information



Supporting Information

Supplemental Movie 1

Supplemental Movie 2

Supplemental Movie 3

Supplemental Movie 4

Supplemental Movie 5

Supplemental Movie 6

Supplemental Movie 7

## Data Availability

All supporting data are available in the supplementary section or from the corresponding authors.
