# Supplementary material for: A Pill That Prints‐An Ingestible Bioprinter for Non‐Invasive Structured Bioink Deposition
Source: Adv Sci (Weinh). 2025 Sep 26;12(46):e12411. doi: 10.1002/advs.202512411 (PMC12697772; doi:10.1002/advs.202512411)
Supplement: Supplementary file 1 — Supporting Information [file ADVS-12-e12411-s007.docx]

SUPPLEMENTARY

Supporting Information for

**A Pill That Prints - An Ingestible bioprinter for non-invasive structured bioink deposition**

*Sanjay Manoharan _1_* & Vivek Subramanian _1_**

_1_ Laboratory for Advanced Fabrication Technologies, Institute of Electrical and Micro Engineering, École Polytechnique Fédérale de Lausanne (EPFL), Switzerland

*Corresponding author. E-mail: [sanjay.manoharan@epfl.ch](mailto:sanjay.manoharan@epfl.ch); vivek.subramanian@epfl.ch

Contents:

Supplementary Notes S1- Magnetically Modulated Stick-Slip Phenomenon (MDSS)

Figure. S1 to S9

Table S1,S2

References

Captions for Movies S1 to S7

**
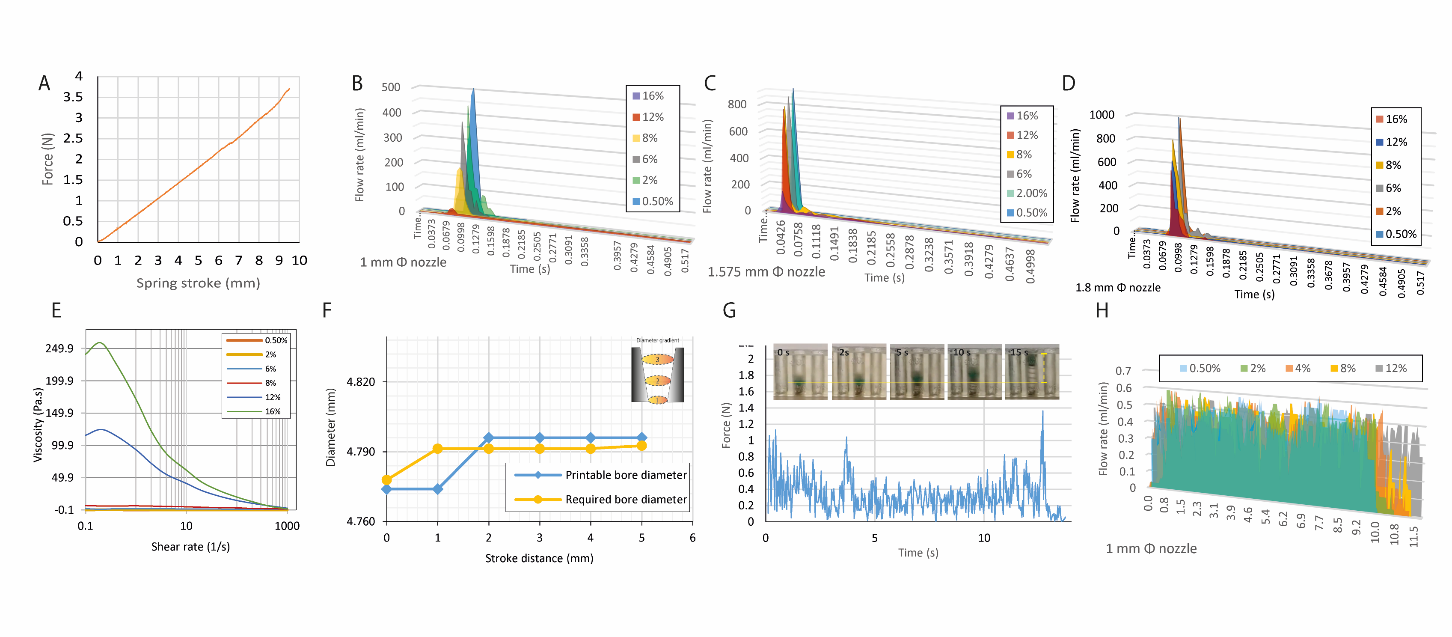
S2 Supplementary figures**

**Figure S1. Bioink flow analysis and sustained spring release through frictional spring dampening**

(A) Force profile of the stainless-steel spring identified via compression testing performed with a Universal Testing system (Instron 6800) (B), (C), (D) Flow profile obtained through various bioink viscosities and nozzle diameters, indicating the persistence of the quick ejection despite the constraints imposed by higher viscosities and smaller nozzle diameters. (E) Viscosity of various alginate concentration bioinks obtained through a rheometer (TA Discovery HR2) (F) Comparison of printable bore diameter and required bore diameter. The goal was to identify the retarding force arising from various bore diameters on the plunger bit and print a barrel with a diameter gradient synchronized to the spring rate in order to achieve sustained spring release could be achieved. There was however a discrepancy between the required bore diameter obtained from the retarding force data and the printable diameter due to the resolution limits of the SLA printer (Phrozen Mini 8K). (G) Force exerted by the plunger when placed in a diameter gradient resulting in a 15s sustained release. (H) Flow profile of various bioink viscosities through 1mm nozzle diameter in response to a diameter gradient, resulting in a 11.5s sustained release. The reduction in release time between G and H can be attributed to the lubricating effect of the bioink at the plunger-barrel interface.


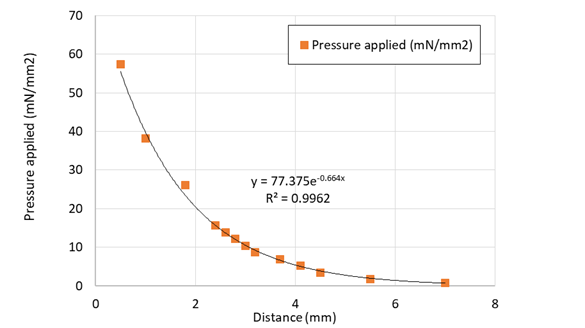


Figure. S2. Pressure applied on hemorrhages as a function of AM-EM distance.

The pressure applied on hemorrhages was altered by changing AM-EM coupling distance reduction, which increases the normal force, the force was measured through a force gauge (Sauter FH 50 EXT)


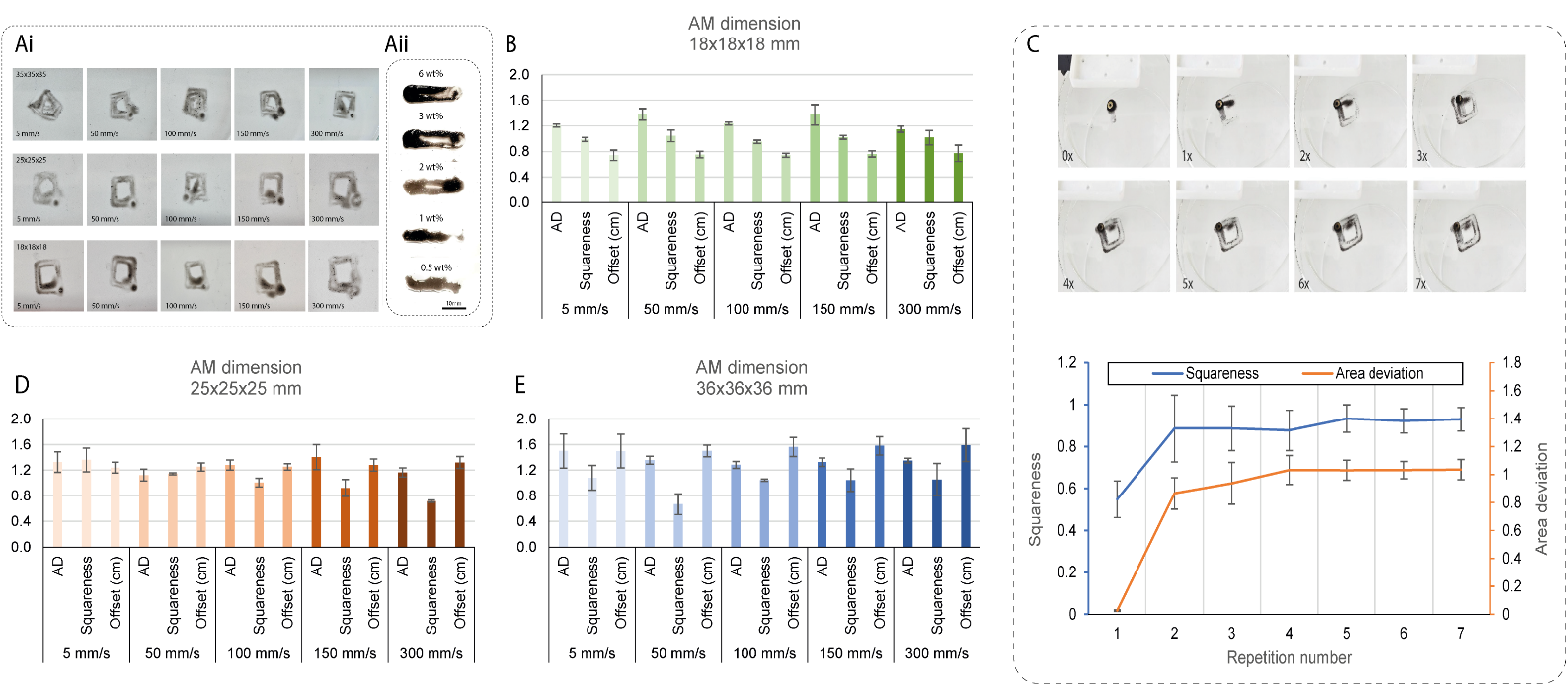


**Figure. S3.** **Print optimization**

**A i-** Representative images of 20mm squares printed with various AM dimensions and AM velocities **ii-** Test straight line prints showing bioink patterns formed using various alginate concentrations (0.5–6 wt%) with 3 wt% yielding optimal balance between flowability, shape integrity and biocompatibility for daub-based deposition.**B,D,E** Plots comparing the print characteristics of the squares and the offset corresponding to various AM dimensions and velocities. **C** Plot depicting the increase in print desirability via squareness with the number of trajectory repetitions and its corresponding experimental images indicating that the 5 trajectory repetitions is ideal for good print fidelity.


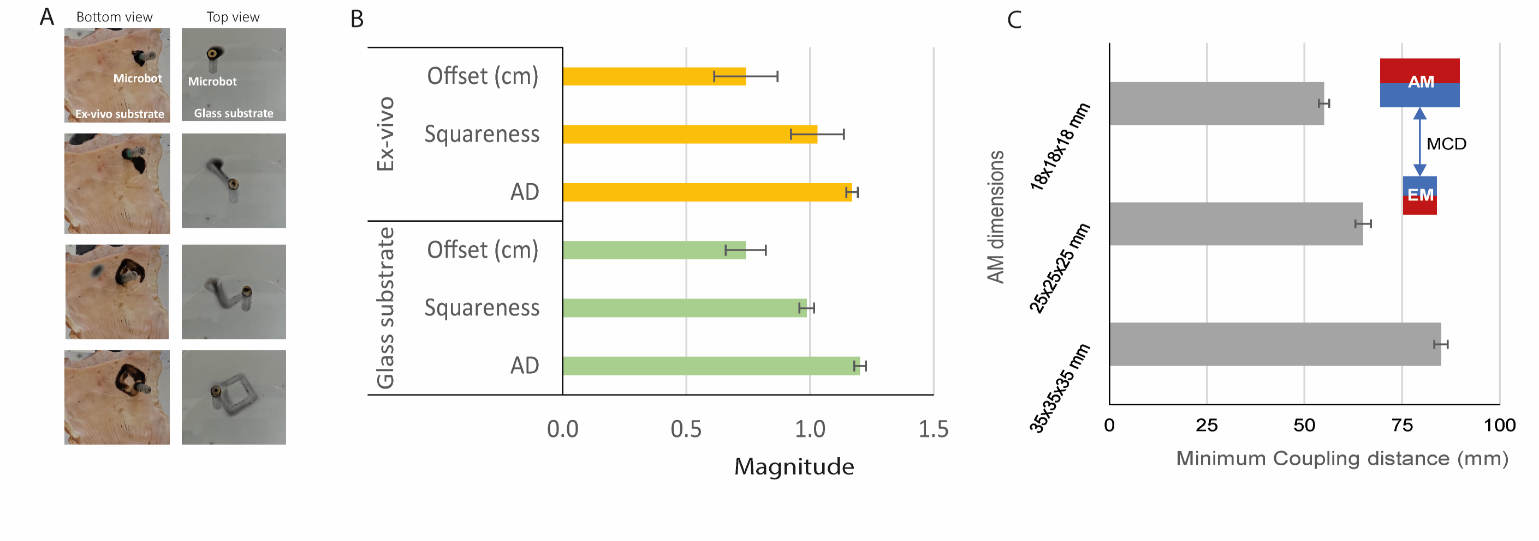


**Figure. S4. Comparison of printing on glass and ex-vivo substrates**

**A** Representative images of 20mm squares printed on ex-vivo porcine gastric tissue and glass with various same control parameters **B** Print characteristics comparison between glass and ex-vivo substrate **C** Minimum distance between the AM-EM required to achieve magnetic coupling


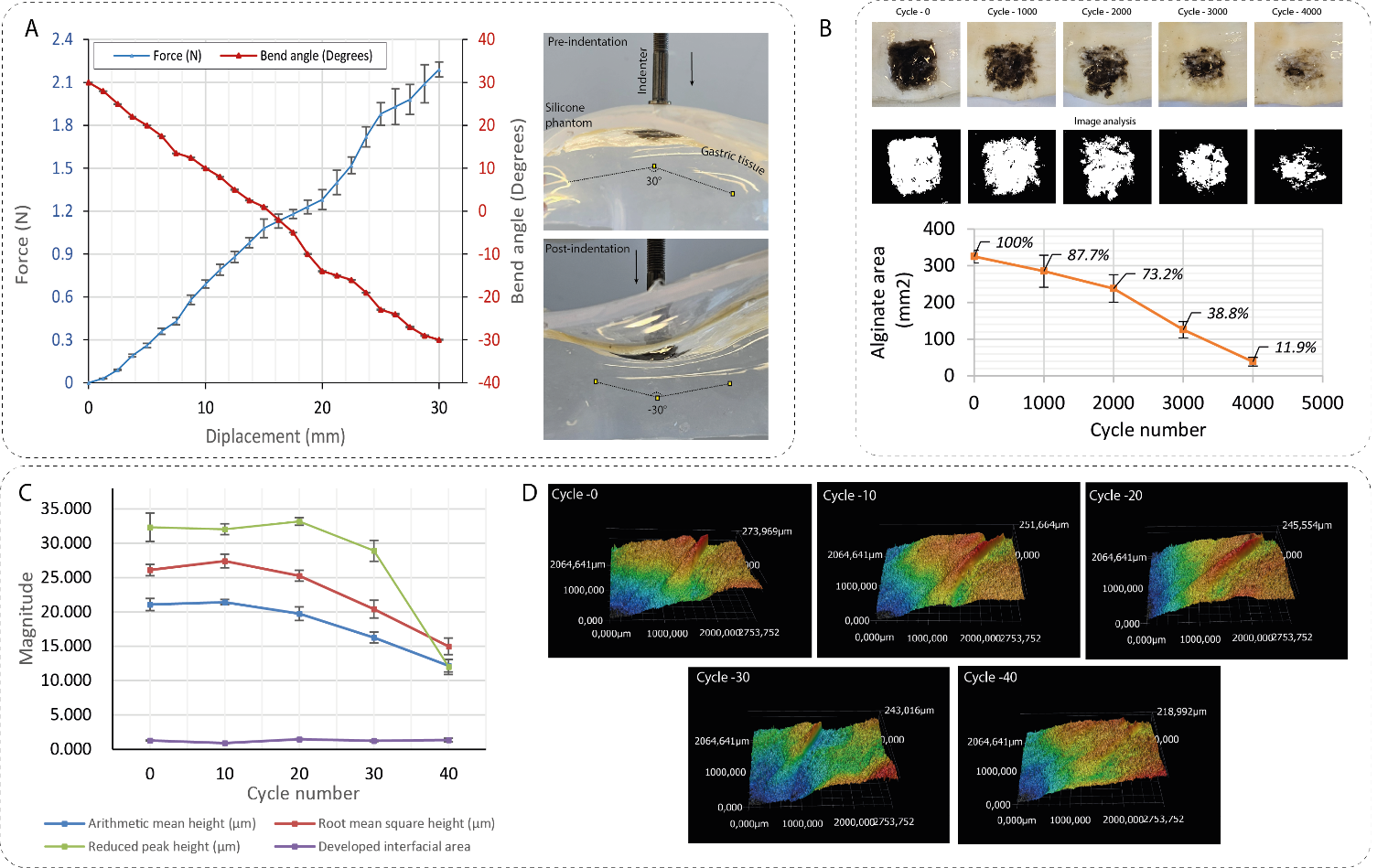


**Figure. S5. Assessment of alginate construct stability under cyclic mechanical loading.
A** Force profile of the indentation that causes the substrate to undergo cyclic bending of 30 degrees to simulate peristalsis. **B** Visual comparison and corresponding quantification of alginate construct degradation from cycle 0 to 4000 under simulated gastric conditions, illustrating progressive erosion patterns from mechanical stress **C** MEDS was actuated over ex vivo tissue in a linear reciprocating motion, with each forward and backward pass counted as one cycle. These trends collectively indicate that repeated capsule motion induces surface flattening and peak reduction consistent with microstructural compression, rather than material removal or abrasive damage. The stable developed interfacial area further supports the absence of contact-induced surface texturing or tearing. **D** Confocal topography maps of the abraded region across 5 cycles show gradual loss of micro texture and topographical flattening. Each heatmap reflects 3D surface deformation localized to the abrasion path.


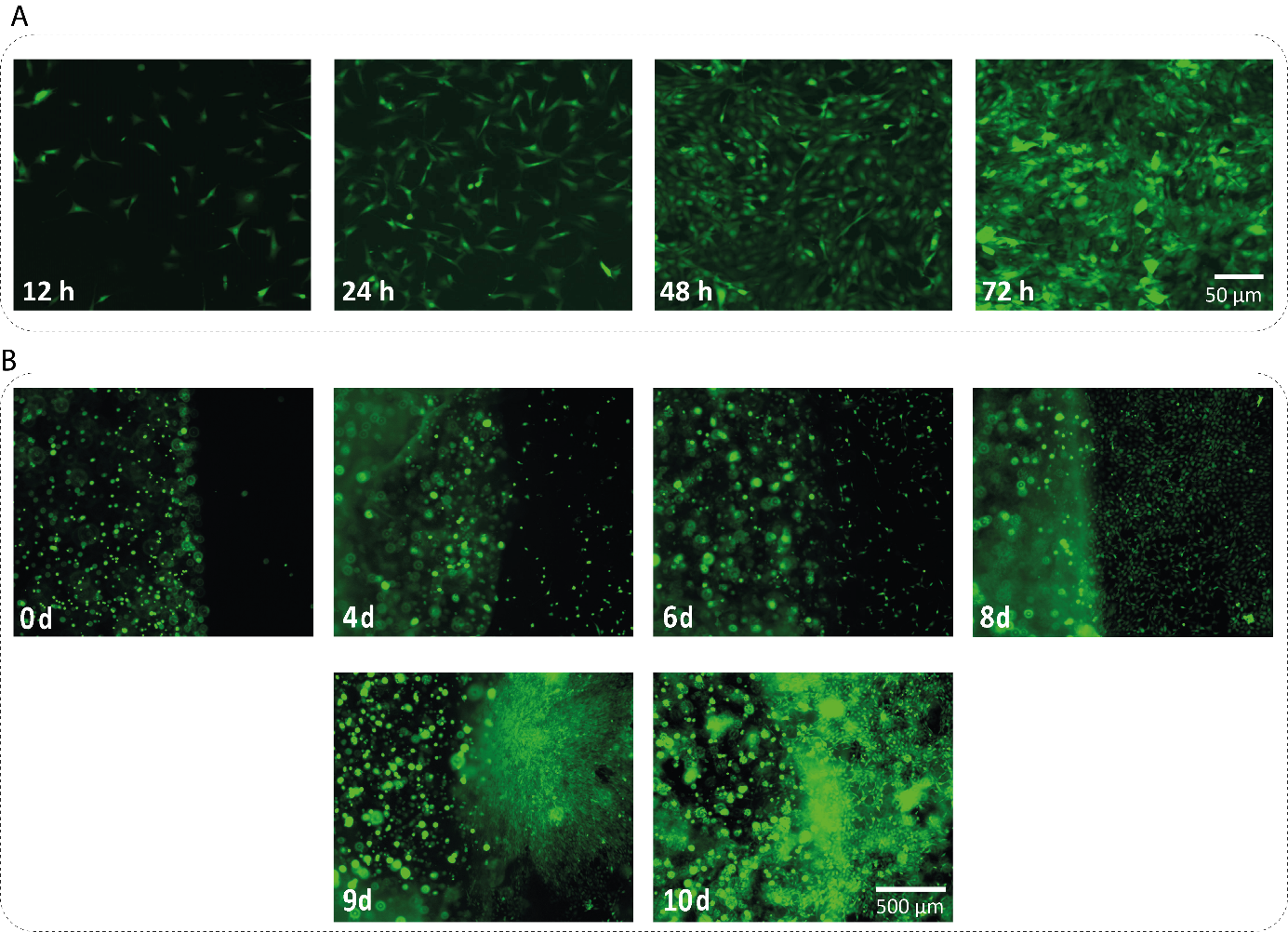


**Figure. S6. Fluorescence Microscopy images of cell proliferation after release**

**A**  Control cell proliferation time course over 3 days. Cells show ideal attachment, spreading, and spindle morphology in standard culture conditions **B** The cells released following the degradation of calcium crosslinks in the hydrogel exhibited normal proliferation signifying reversible mitotic activity. This emphasizes the potential for these hydrogel constructs to act as a cell supply reservoir to deliver cells for healing to wound sites.


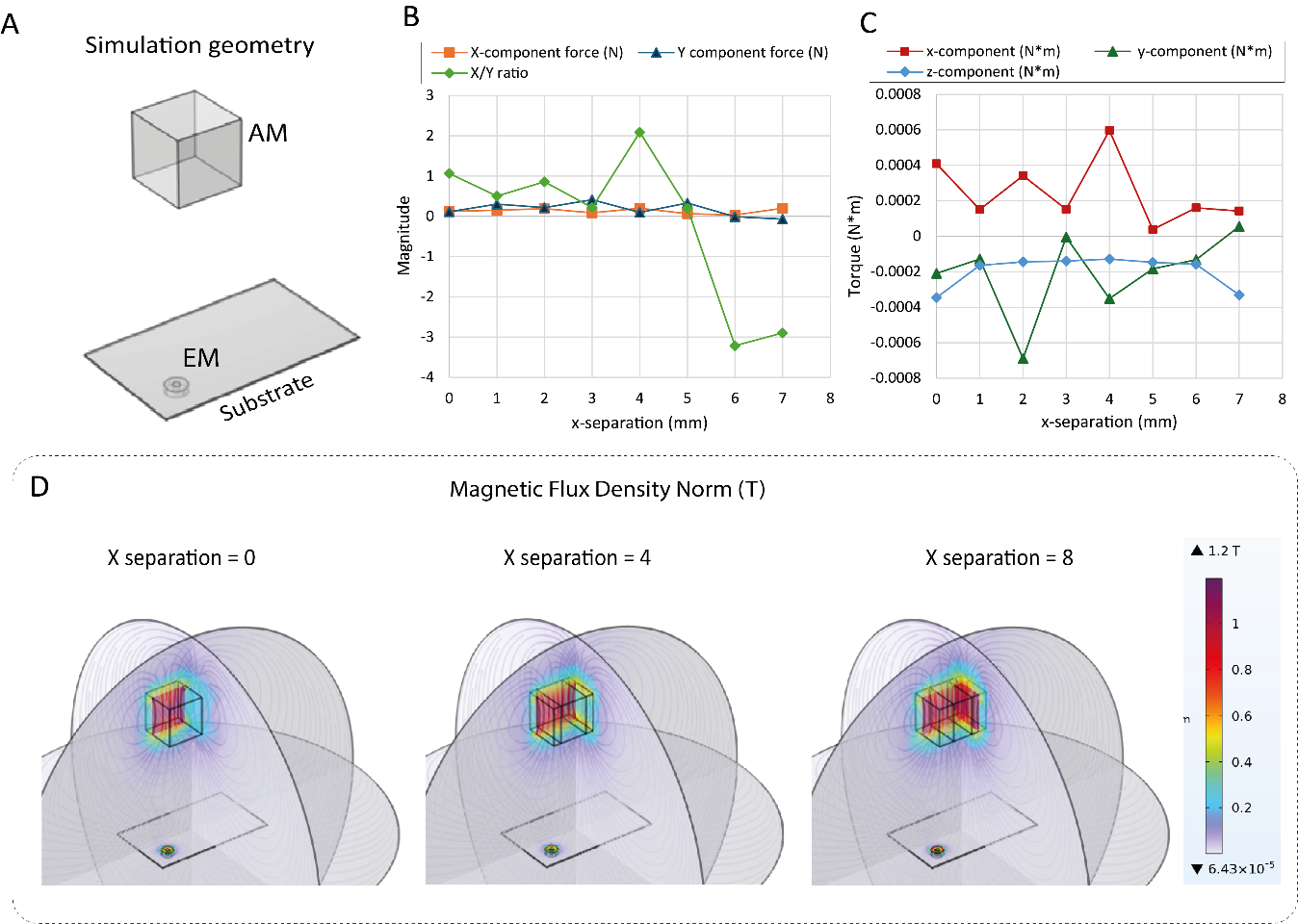


**Figure.S7: Magnetics simulation setup and results with COMSOL MFNC module**

**A** Simulation geometry of the AM-EM setup **B** Plot of change in force components, ratio of the force components **C,D** Plot of change in torque components and its corresponding magnetic flux density visualization at various x-separations


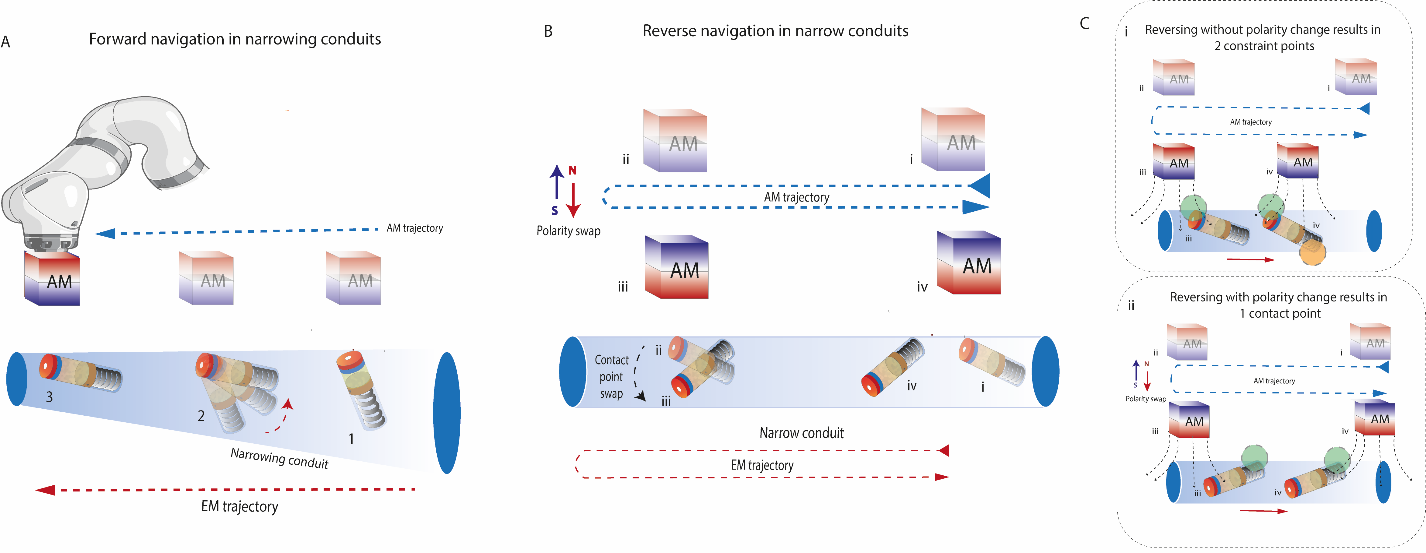


**Figure.S8: Mechanism of forward and reverse navigation in in tight spaces**

**A** Illustration of forward navigation in tight spaces. A narrowing conduit automatically constraints the printer and changes its angle of attack thereby promoting smooth forward navigation until the space is too tight to proceed **B** Illustration of a strategy for smooth reverse navigation in tight spaces involving AM polarity swap. Swapping the AM polarity enables smooth reverse navigation due to shallow angle of attack **C** Illustration of the mechanism behind smooth reverse locomotion arising from AM polarity swap. In spacious areas, due to unimpeded movement of MEDS, a polarity swap will not be required. angles of the polarity-unchanged, reversing AM**. C-i** In space-constrained regions, a steeper flux angle offered by the reversing AM and the EM’s attempts to align with the steep south pole flux lines results in a high angle of attack. This led to two constraining or contact points at the top and the bottom which prevent and brake the reverse motion. **C-ii** Contrastingly if the polarity is swapped by rotating the AM along the magnetization axis, the EM’s south pole can align easily with the flatter flux angles of the reversing AM’s north pole via contacting its bottom with the ceiling. This promotes a shallow angle of attack and hence a smoother reverse motion.

S1. Supplementary Text

**Magnetically Modulated Stick-Slip Phenomenon (MDSS)**

A surface dragged object does not enjoy smooth lateral motion but rather exhibits a phenomenon referred to as stick-slip. This is because the initial push is in order to overcome the static friction or sticking regime; when the driving force overcomes static friction it is in the slipping regime. Then because of local increments in force, it slows down because of micro-scale to atomic-scale vibrations leading to another sticking event. In this case, the force fluctuation of the dragged object can be explained mechanically.


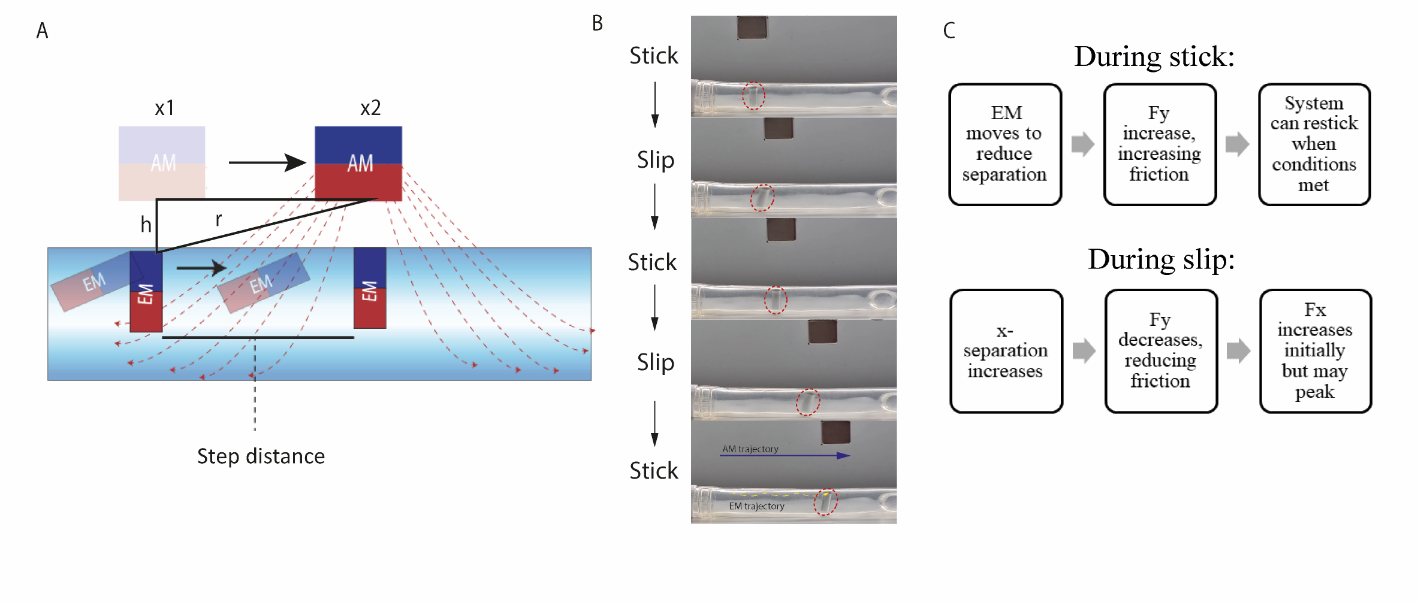
However, in Magnetically Modulated Stick-Slip (MMSS) phenomenon the force varies axially depending on the relative position of the AM to EM. When the EM is being dragged by the AM, the force is not just a simple horizontal pull; instead, it involves a complex interplay of horizontal and vertical magnetic force components that varies with the EM-AM relative positions as shown in Supplementary Figure.

**Figure. S9. Magnetically modulated stick slip phenomenon explanation**

**A, B** Illustration of the MMSS process and its experimental demonstration with an empty magnetic capsule **C** Schematic of the processes involved in each stick or slip cycle

The key components of this magnetically modulated stick-slip model are-

1. F_y_ affects the normal force and thus friction while F_x_ provides the driving force.
2. The force ratio in x and y axis, F_x_/F_y_ experienced by the EM changes with AM position, even without traditional friction.
3. As the AM moves, both force magnitude and direction change.
4. The dragged magnet experiences periodic changes in net force that can cause stick-slip behavior.

The stick-slip arises due to -

1. There is EM falling behind the AM, which increases the horizontal force component.
2. This leads to a sudden 'catch up' motion given by the step distance as shown in Supplementary Figure.8.
3. As it catches up, the vertical force component increases leading to another stick period.

This can occur simultaneously with traditional friction-based stick-slip, where the traditional friction component becomes higher when the magnets are vertically aligned and becomes lower with horizontal x-separation. In these systems, the traditional friction and magnetic forces can either synergistically reinforce each other resulting in aggravated stick-slip or work against each other for smooth movement.

The mathematical model described below is intended as a tool to explain the empirical observations

**Model Variables**

***x₁*** = position of AM; ***x₂*** = position of EM; ***h***= vertical separation between magnets; ***μ_s_*** = static friction coefficient; ***μ_k_*** = kinetic friction coefficient; ***m*** = mass of driven magnet; ***V*** = velocity of AM; ***r*** = distance between magnets = √((x₁-x₂) ² + h²)

***Fx/Fy = x_1_-x_2_/h***, varies continuously with position, creating natural oscillations between stick and slip states.

1. **Magnetic forces**

*F_total_ = k/r³*

Where, *k = (μ₀M₁M₂)/(4π)*

x-force component, the horizontal pulling or driving force is given by,

*F_x_ = F_total_ ((x₁-x₂)/r^4^)*

y-force component, the vertical attraction force is given by,

*F_y_ = F_total_ (h/r) = kh/ r^4^*

1. **Friction Force**

*F _friction_ = μ N i.e.,*

*μ_s_(Fy - mg),* if static

*μ_k_ (Fy - mg) (v),* if kinetic in velocity-dependent systems

1. **Normal Force**

*N = Fy -mg*

For the magnet to be pulled up away from the bottom of the lumen,

*Fy > mg* ***⇒*** *Fy_min_ = kh/r^4^_max_ > mg*

1. **Equations of Motion**

**i) During Stick Phase (Stick occurs when: F_x_ < F_y_μ_s_):**

*dx₂/dt = V* (EM moves with driving force)

*x₁ = Vt* +*x_2_​(0)* **⇒** *x₁ = Vt* (Position of AM at time zero)

**ii) During Slip Phase (Slip occurs when: F_x_ > F_y_μ_s_):**

Although, kinetic friction is independent of speed in most cases, in certain real-world scenarios, such as fluid dynamics and damped systems, friction is often described as being dependent on velocity.

Hence,

*ma=F_x_-F_friction_* ⇒ *m(d²x₂/dt²) = F_x_ - μ_k_N(dx₂/dt)*

*m(d²x₂/dt²) = F_x_ - μ_k_N(dx₂/dt)*

wher(E)

N = mg - kh/r³

**iii) Transition Conditions**

***Stick → Slip:***

*Fx > μ_s_ (mg - Fy) ⇒ k(x₁-x₂)/r^4^ > μ_s_(mg - kh/r³)*

***Slip → Stick:***

*dx₂/dt = V* and *F_x_ ≤ μ_s_ (mg - Fy)*

*m(d²x₂/dt²) = k(x₁-x₂)/r^4^ - F_friction_*

This creates a unique situation where stick-slip can occur even with constant friction coefficients, purely due to the changing magnetic force geometry. When both mechanisms are present, they can either reinforce each other (creating more pronounced stick-slip) or work against each other (potentially smoothing the motion). This dual nature makes magnetic stick-slip more complex and potentially more controllable than traditional friction-only stick-slip.

Hence, the pre-extrusion experimental optimization to avoid spatiotemporal discrepancies focused on fine-tuning(G)

1. **Minimum coupling distance *or* Maximum AM-EM Separation:**

*r_max_ = (kh/mg) ^1/3^*

*x_max_ = √ (r_max_² - h²)*

1. **Optimal Operating Rang(E)**

x_separation_ < x_max_ to prevent falling

x_separation_ > x_min_ to allow motion

**Effect of a lubricating surface on MMSS**

When a lubricant in the form of bioink is introduced between the EM and the substrate, it alters the EM-substrate interaction in the following ways –

**Reduction in Friction Coefficients**

The continuous presence of bioink reduces the μ_s_ and μ_s_ due to its lubricating properties.

A lower μ_s_ translates to fewer stick events since the threshold for sticking is now harder to attain. Mathematically, in the transition Stick → Slip equation “|Fx| > μ_s_ (mg - Fy)”, Fx easily exceeds the RHS. Further, when bioink is introduced, it shifts the system towards the high-Stribeck-number, hydrodynamic lubrication regime. In this high-Stribeck regime, stick-slip behavior becomes highly unlikely or impossible, as the lubrication effectively prevents any sticking due to the continuous fluid film separating the surfaces. On the other hand, in the sustained extrusion condition, the presence of an intermittent bioink availability for the EM leads to an inconsistent bioink interface, which merely prolongs the slip phase.

**Reduction in Normal Force**

The lubricating bioink reduces the contact area between the EM and substrate by shielding it from contact with substrate asperities.

**Damping Effect of the Bioink**

The bioink acts as a damping medium, absorbing some of the kinetic energy spent on oscillations in the EM navigation, which in turn reduces the stick and slip phases following this-

m(d²x₂/dt²) + c(dx₂/dt) = Fx - μkN(dx₂/dt)

where, c represents the damping effect of the bioink.

Table S1. Comparison of MEDS with other in-situ bioprinter technologies

| **Bioprinter** | **Dimensions** | **Key Features** | **Applications** | **Performance** | **Advantages** | **Disadvantages** |
| --- | --- | --- | --- | --- | --- | --- |
| **MEDS** | Φ = 6 mm, Length = 14 mm, weight ≈ 28 mg with bioink | NIR-activated, magnetically steerable, tetherless, electronics-free | GI tract treatments (ulcers, hemorrhages) | **Nozzle Φ =** 1mm, stroke width 6.5 mm  Average area deviation on flat substrates < 17.64 mm²  **Operating distance:** 55mm (18mm AM) to 85mm (35mm AM)  **Working volume: 5,747 mm³ (**hemispherical, radius 14mm)  **Angular resolution:** Pitch 3.23 ± 0.59°, Yaw 3.67 ± 0.73°, Roll 12.33 ± 1.38°  Complete 360° rotational freedom | **Access Method:** Oral ingestion → Oral retrieval  **Control:** Single external permanent magnet  **Operation:** Electronics-free, tetherless  **Activation:** External NIR triggering through intact tissue  **Capability:** Repositioning and redeployment potential  **Clinical Model:** Medication-like administration vs. procedure-based intervention  **Anatomical Reach:** Potential for full GI reach | Limited bioink capacity, minimal fidelity, limited to single print layer |
| **Mechatronic endoscope-like bioprinter** ^[19]^ | Φ ≈ 20 mm (4× wider than MEDS) | Installed on generic endoscope, mechatronic control | Gastric repair | Average printed fiber diameter = 500 μm  Lattice structure deviation: 1.18 mm ± 0.43 mm  Planar circle deviation: 0.67 mm ± 0.15 mm  Theoretical: 0.24° step angle<br>Specific axis ranges: Not reported | **Access Method:** Endoscopic insertion  **Integration:** Future potential for conventional endoscope integration | **Access:** Requires endoscopic access  **Size:** Bulkier than traditional endoscopes  **Flexibility:** Poor due to stiff linkage and rigid components  **Control:** Complex mechatronic assembly |
| **F3DB-Flexible in-situ 3D Bioprinter** ^[20]^ | Φ = 20 mm (4× wider), Length = 50 mm (5× longer), weight ≈ 100 g (3571× heavier) | Tethered system, hydraulic-driven swiveling printhead | Gastric/colonic printing, dissection, electrosurgery | Nozzle Φ = 0.5-0.9mm  Printing Accuracy (MSE) < 0.083 mm²  Hydraulic swivel control  **Specific resolution:** Not reported | **Access Method:** Endoscopic insertion with specialized channels  **Functionality:** Multifunctional, all-in-one endoscopic tool potential | **Control:** Complex hydraulic control system  **Imaging:** Lack of integrated imaging  **Compatibility:** Poor compatibility with standard imaging techniques  **Operation:** Tethered system |
| **FSCR - Ferromagnetic Soft Catheter Robot** ^[10]^ | Φ = 4–7 mm, Length = 80–110 mm (8-11× longer than MEDS) | Magnetic actuation, compliant nozzle | Laparoscopic printing | Nozzle Φ = 0.6mm  Best resolution = 0.53 mm  Effective on flat and curved substrates  Bending: Up to 30% deflection ratio | **Access Method:** Laparoscopic insertion  **Accuracy:** High accuracy on curved surfaces  **Invasiveness:** Minimally invasive | **Control:** Four permanent magnets required (4× more complex  **Scalability:** Limited  **Platform:** Small printing platform size  **Access:** Limited to laparoscopically accessible regions |
| **Handheld Skin Printer** ^[18]^ | <0.8 kg, Large-format printhead, portable | Lightweight, integrated design | Wound healing, skin grafts | Not specified  Manual operation (no automated angular control) | **Access Method:** External application  **Portability:** Portable design  **Control:** Local control over biomaterial composition  **Operation:** Easy to operate | **Application:** Limited to surface applications only  **Dependency:** Operator skill-dependent  **Scope:** Cannot access internal anatomy |

**Key Differentiating Metrics:** MEDS achieves superior performance across critical evaluation criteria: completely non-invasive access (score 0) via oral route versus moderate invasiveness for endoscopic systems (score 2) and full surgical access for laparoscopic approaches (score 3); simplest control architecture using 1 external magnet plus NIR versus 4× more complex multi-magnet systems (FSCR) or complex mechatronic/hydraulic assemblies; complete GI tract accessibility through natural pathways compared to upper GI limitations (endoscopic), abdominal cavity restrictions (laparoscopic), or surface-only applications (external); unique tetherless working volume of 5,747 mm³ with hemispherical 360° accessibility versus linear/constrained workspaces of tethered systems; competitive angular control with pitch resolution of 3.23 ± 0.59°, yaw resolution of 3.67 ± 0.73°, and roll resolution of 12.33 ± 1.38° while maintaining complete rotational freedom impossible with cable-constrained devices; and significant miniaturization with endoscopic systems being 4× wider, F3DB being 4× wider/3.6× longer/3571× heavier, FSCR being 5.7-7.9× longer, and handheld devices being 28,571× heavier. Additionally, MEDS demonstrates operational capabilities including omnidirectional access, unlimited rotation, independent positioning without path constraints, and complete inversion capability, functionalities fundamentally impossible with tethered architectures due to physical cable limitations.

Table S2. Comparison of MEDS with other smart capsule technologies

| **System** | **Core Idea** | **Dimensions** | **Limitations vs. MEDS** | **MEDS Advantages** |
| --- | --- | --- | --- | --- |
| **PILLSID Refill capsule (implant + ingestible cartridge)**^[35]^ | Capsule docks magnetically to an implanted reservoir and refills drug depot | Implant device = 78 x 63 x 35 mm  Refill capsule = Ø 12 mm × 28 mm  8.0× MEDS volume | Requires permanent implant; only sustains systemic drug dosing | MEDS is implant-free, self-contained, suited for acute interventions |
| **RoboCap**^[88]^ | Active capsule clears mucus & mixes fluid to enhance local drug absorption | 9.9x26 mm  ~5.09× MEDS volume | Mainly improves absorption; no active positioning or printing | MEDS offers precise navigation + localized structured bioink patterning |
| **Cephalopod Jetting Device**^[33]^ | Biomimetic ingestible device expels liquid payload by jet propulsion | Ø 10.8 mm × 11.8 mm  2.73× MEDS | Release is bulk, uncontrolled spray; no site targeting | MEDS allows spatiotemporally controlled extrusion and bioink patterning |
| **SOMA Capsule**^[32]^ | Self-orienting capsule injects compressed spring-loaded, drug “millipost” into gastric tissue | Ø 9 mm × 15 mm  ~2.41×MEDS | One-time injection, no navigation, designed for systemic delivery | MEDS provides multi-step, retrievable, magnetically guided deposition of bioinks. controlled extrusion + patterned deposition without puncture |
| \| **Microneedle Ingestible Device**^[34]^ \| \| --- \|  \|  \| \| --- \|  \|  \| \| --- \| | Capsule with spring-loaded microneedles injects drug across gastric mucosa | Module OD 13 mm (length not stated) | Single-shot bolus injection; puncture risk; no active navigation | MEDS provides controlled extrusion + patterned deposition without puncture |
| **MagCaps**^[31]^ | Multifunctional magnetically actuated capsule with integrated soft magnetic valve for drug release and sampling | ~10 mm diameter or more (multiple models)  ~2.5×MEDS | Larger than MEDS; needs external coil arrays | Wireless, biofabrication system for structural intervention and soft tissue repair |

Key Differentiating Metrics: Unlike existing ingestible systems that act only as drug delivery vehicles, MEDS is the first pill-scale, tetherless biofabrication platform for the GI tract. Measuring just 6 mm × 14 mm and 28 mg, it is 2.4 -8× smaller than contemporaries and requires only a single external magnet for navigation with NIR-triggered extrusion, avoiding complex tethers or multi-magnet arrays. Beyond payload release, MEDS achieves in situ bioink printing, enabling sealing, scaffolding, and localized repair. In short, drug release is pharmacotherapy, whereas bioink printing is structural and regenerative therapy, uniquely positioning MEDS for acute GI interventions.

S3 supplementary movies

Movie S1.

Simplified animated video abstract of MEDS deployment portraying the ingestion, pre-extrusion positioning, patterning and expected healing outcomes

Movie S2.

In-vitro demonstration of the hemostatic sealing process

Movie S3.

In-vitro waypoint navigation test using a dot grid

Movie S4.

In-vitro demonstration of pre and post-extrusion positioning in a gastric phantom

Movie S5.

Printing on ex-vivo substrates

Movie S6.

Printing spirals inside the perimeter of a medium sized ex-vivo ulcer

Movie S7.

X-ray fluoroscopy guided positioning, extrusion and patterning
